# Revealing taxon-specific heavy metal-resistance mechanisms in denitrifying phosphorus removal sludge using genome-centric metaproteomics

**DOI:** 10.1186/s40168-021-01016-x

**Published:** 2021-03-22

**Authors:** Yuan Lin, Liye Wang, Ke Xu, Kan Li, Hongqiang Ren

**Affiliations:** State Key Laboratory of Pollution Control and Resource Reuse, School of the Environment, Nanjing University, N.O.163, Xianlin Avenue, Nanjing, Jiangsu People’s Republic of China

**Keywords:** Metagenomics, Metaproteomics, Sludge, Heavy metal resistance

## Abstract

**Background:**

Denitrifying phosphorus removal sludge (DPRS) is widely adopted for nitrogen and phosphorus removal in wastewater treatment but faces threats from heavy metals. However, a lack of understanding of the taxon-specific heavy metal-resistance mechanisms hinders the targeted optimization of DPRS’s robustness in nutrient removal.

**Results:**

We obtained 403 high- or medium-quality metagenome-assembled genomes from DPRS treated by elevating cadmium, nickel, and chromium pressure. Then, the proteomic responses of individual taxa under heavy metal pressures were characterized, with an emphasis on functions involving heavy metal resistance and maintenance of nutrient metabolism. When oxygen availability was constrained by high-concentration heavy metals, comammox *Nitrospira* overproduced highly oxygen-affinitive hemoglobin and electron-transporting cytochrome c-like proteins, underpinning its ability to enhance oxygen acquisition and utilization. In contrast, *Nitrosomonas* overexpressed ammonia monooxygenase and nitrite reductase to facilitate the partial nitrification and denitrification process for maintaining nitrogen removal. Comparisons between phosphorus-accumulating organisms (PAOs) demonstrated different heavy metal-resistance mechanisms adopted by *Dechloromonas* and *Candidatus* Accumulibacter, despite their high genomic similarities. In particular, *Dechloromonas* outcompeted the canonical PAO *Candidatus* Accumulibacter in synthesizing polyphosphate, a potential public good for heavy metal detoxification. The superiority of *Dechloromonas* in energy utilization, radical elimination, and damaged cell component repair also contributed to its dominance under heavy metal pressures. Moreover, the enrichment analysis revealed that functions involved in extracellular polymeric substance formation, siderophore activity, and heavy metal efflux were significantly overexpressed due to the related activities of specific taxa.

**Conclusions:**

Our study demonstrates that heavy metal-resistance mechanisms within a multipartite community are highly heterogeneous between different taxa. These findings provide a fundamental understanding of how the heterogeneity of individual microorganisms contributes to the metabolic versatility and robustness of microbiomes inhabiting dynamic environments, which is vital for manipulating the adaptation of microbial assemblages under adverse environmental stimuli.

Video abstract

**Supplementary Information:**

The online version contains supplementary material available at 10.1186/s40168-021-01016-x.

## Background

Heavy metal pollution is consistently a critical issue in many parts of the world, mainly attributed to their intensive consumption [1, 2]. Wastewater treatment plants (WWTPs) inevitably become the source and sink of these toxic heavy metals [3]. Denitrifying phosphorus removal sludge (DPRS) is a typical artificial ecosystem widely adopted in biological processes (e.g., anaerobic/anoxic/aerobic process) by numerous WWTPs worldwide [4, 5]. Due to the presence of anaerobic and anoxic phases, DPRS exhibits greater nitrification capacity and phosphorus removal performance than activated sludge [6]. But heavy metals can cause dysfunctions of proteins and inhibit microbial activities such as nitrification [7, 8] and eventually attenuate the treatment efficiency [5, 9]. Thus, a better understanding of the heavy metal-resistance strategies adopted by DPRS microbiomes might ultimately improve their robustness for nutrient removal.

Current knowledge of mechanisms for microbes to resist heavy metals is mainly based on pure cultures [10]. Specifically, metal-resistance genes (MRGs) are those having experimentally evidenced merits to decrease the carrier’s susceptibility to metals [11]. Besides, microbes are shown to relieve heavy metals’ toxicity by producing extracellular polymeric substance (EPS) [12, 13], outer-membrane vesicles [14], inorganic polyphosphates (polyP) [15], and metallophores [16, 17], or regulating outer membrane permeability [18]. On the one hand, MRGs are mainly retrieved from studies performed on model organisms such as *Escherichia coli*, *Pseudomonas aeruginosa*, and *Saccharomyces cerevisiae* [10, 11]. Those model organisms are rare in sludge systems and can hardly represent the core populations, especially the functional microbes (e.g., *Nitrospira* and *Candidatus* Accumulibacter), in DPRS [4]. In addition, due to the presence of interspecific interactions in complex communities, microbes behave differently from those in pure cultures [19, 20]. Therefore, mechanisms found in pure cultures are not necessarily widespread or used in complex communities [21]. On the other hand, functional variances observed at the community scale may not precisely reflect the metabolic changes of individual microbes. The ambiguity causes difficulty in identifying heavy metal-resistance mechanisms of specific taxa. However, present studies concerning heavy metal resistance in microbial communities mainly focused on soils or sediments and mostly characterized functional changes in a community-resolved manner [22–25]. Hence, an investigation specific in DPRS and also at a genome-scale resolution is pivotal.

The response of individual taxa to environmental stimuli in a complex community can be characterized using genome-resolved multiomics [26]. For instance, genome-centric metatranscriptomics has been successfully applied for identifying the most responsive microbes to exogenous stimuli in anaerobic digesters [27]. In parallel to metatranscriptomics, metaproteomics reveals a further direct functional profile of the microbial community under a given condition [28]. Using genome-guided metaproteomics provides the opportunity to link taxonomic identities with actively expressed functions involved in heavy metal resistance.

To get a comprehensive understanding of the heavy metal-resistance mechanisms, we chose Cr (VI), Ni (II), and Cd (II) as representatives of heavy metals. They are ubiquitous in wastewater and highly toxic but distinctive in properties in terms of oxidation state, ionic radius, and polarizability [10]. We monitored the nutrient removal performance, microbial activities, EPS content, and heavy metal distribution in DPRS using lab-scale bioreactors treated with different heavy metals. Metagenome-assembled genomes (MAGs) were obtained from sludge sampled at all stages in all reactors. Then, we applied genome-centric metaproteomics to characterize the functional changes of individual microbes under the most intensive pressure. Given the importance of nitrogen and phosphorus removal, we mainly focused on the resistance mechanisms of key taxa involved in nutrient removal. Nevertheless, other microbes that contributed significantly to the heavy metal resistance were also characterized.

## Methods

### Setup and operation of bioreactors

We set up a parent sequencing batch reactor (20 L) for the acclimation of DPRS. Seeding sludge was obtained from a full-scale WWTP in Danyang, China. The parent reactor was fed with synthetic wastewater (see Additional file 1: Text S1 for the detailed composition) and run in four anaerobic/anoxic/aerobic cycles (detailed operating strategy is in Additional file 1: Table. S1) per day. After 240 days of acclimation, the nitrogen and phosphorus removal rate was stable at 78±7% and 95±3%, respectively.

Sludge from the parent reactor was evenly distributed into four daughter reactors whose working volume was 4 L. The operating strategy for daughter reactors was identical to that of the parent reactor. We chose heavy metals’ concentrations in the ranges of their presence in real wastewater, according to the recent reports (Additional file 1: Table S2). R_Cd_, R_Ni_, and R_Cr_ were fed with synthetic wastewater containing 0.1 mg/L cadmium chloride (CdCl_2_), 0.1 mg/L nickel chloride (NiCl_2_), and 0.2 mg/L sodium chromate (Na_2_CrO_4_), respectively. All reagents were purchased from Sigma-Aldrich (Shanghai branch, China). The concentration of heavy metal was elevated by an order of magnitude after the reactor performed steadily (the relative deviations of both total nitrogen and total phosphorus removal rates kept below 20%) for at least 48 days. Meanwhile, R_CK_ was run as control by feeding raw synthetic wastewater. For brevity, the operating period under low-concentration (0.1 mg/L for Cd and Ni while 0.2 mg/L for Cr), medium-concentration (1 mg/L for Cd and Ni while 2 mg/L for Cr), and high-concentration (10 mg/L for Cd and Ni while 20 mg/L for Cr) heavy metal exposure was noted as the low-, medium-, and high-pressure stages, respectively.

The concentrations of nitrogen, phosphorus, and corresponding heavy metal in the influent and effluent were analyzed every 2 days. Specific oxygen uptake rate (SOUR), microbial viability, and EPS content were analyzed three times a month. Details for analytical methods are described in Additional file 1: Text S2, and all tests were carried out in triplicate for each analytical event. Sludge samples (*n*=3 for each reactor per sampling event) for DNA and protein extraction were collected at the end of the aerobic phase. Sampling was performed daily at the last 3 days of each stage as replicates, and all samples were stored at −80 °C after quenching in liquid nitrogen.

### Metagenome assembly, binning, and taxonomic classification

DNA was extracted using FastDNA® SPIN Kit for Soil (MP Biomedicals, Irvine, California USA). Shotgun sequencing was performed on HiSeqXten platform (Illumina, San Diego, California, USA) with 2 × 150-bp read length. Quality control of raw reads was performed using Trimmomatic [29] (v0.39). Qualified reads from all samples were co-assembled into contigs by MEGAHIT [30] (v1.2.9). Binning was performed using MaxBin2 [31] (v2.2.6), Metabat2 [32] (v2.13), and CONCOCT [33] (v1.1.0) following by dereplication and aggregation of the similar bins by MetaWRAP [34] (v1.3). Potential contaminative contigs in each genome were removed using RefineM (v0.1.2) according to Parks et al. [35]. Then, reassembly by MetaWRAP further improved the bins’ completeness and reduced their contamination. The quality of retrieved metagenome-assembled genomes (MAGs) was estimated by checkM [36] (v1.0.13). Finally, we obtained 82 high-quality and 321 medium-quality MAGs that met with the MIMAG standard [37] and had a quality score > 45 (quality score=completeness-5×contamination) [38]. The average nucleotide identity and amino acid identity between a pair of genomes was calculated using orthoANI [39] and compareM [35], respectively. A phylogenetic tree of MAGs was constructed following the UBCG pipeline [40] (v3.0) and visualized using iTOL [41] (v5.0). MAGs were taxonomically classified using GTDB-Tk [42] (v1.02). The corresponding NCBI taxonomy was then converted from the GTDB taxonomy. Details for metagenomic data processing are given in Additional file 1: Text S3.

### Protein extraction, identification, and quantification

Protein extraction was performed using the sodium dodecyl sulfate-phenol method [43] with some modifications (Additional file 1: Text S4). The analysis was performed on a nanoHPLC-MS/MS system consisting of an EASY-nLC 1200 UHPLC connected to a Q Exactive HF-X (Thermo Fisher Scientific, Waltham, MA, USA) operating in data-dependent acquisition mode (see Additional file 1: Text S5 for details).

Protein-coding genes in the metagenome and each MAG were estimated by Prodigal [44] (v2.6.3) in mode “meta” and “single,” respectively. Custom database for protein identification consisted of predicted protein-coding genes and their best matches against NCBI non-redundant protein database (downloaded at 2019/5/4) with *e*-value < 10^−10^, identity > 95%, and coverage > 80% by DIAMOND [45] (v0.9). The spectrum data were processed by Proteome Discoverer 2.2 (Thermo Scientific) against the custom database, and quantification was based on the MS^1^ intensities. The detailed database-searching strategy and Proteome Discoverer’s configuration are described in Additional file 1: Text S6 and Table S3, respectively.

The metaproteome recruitment to the predicted protein-coding genes in MAGs was performed using DIAMOND with *e*-value < 10^−10^, identity > 95%, and coverage > 90%. The detected peptide sequences from that protein were also aligned against the predicted protein-coding gene by DIAMOND for each potential match. Matches with imperfect peptide alignments (< 100% identity or < 100% alignment coverage) were then discarded to filter out potential false positives.

### Functional annotation of MAGs and identified proteins

Well-known functional genes (Additional file 1: Table S4) involving nitrogen [46] and phosphorus [47] removal were retrieved from NCBI using the “Identical Protein Groups” search engine. Only entries ≥ 300 amino acids (except for *amo*ABC, which satisfied ≥ 200 amino acids) were collected. Poorly annotated (putative, probable, or unclassified), ambiguous, and irrelevant results were discarded manually. Qualified sequences were then used as references to retrieve functional genes in MAGs using DIAMOND at *e*-value < 10^−10^, identity > 50%, and coverage > 80%. Meanwhile, conserved domains of the functional genes were searched against an integrated hidden Markov model database [48] by METABOLIC (https://github.com/AnantharamanLab/METABOLIC). Validated matches should be successfully retrieved by both DIAMOND and METABOLIC. MRGs in MAGs were searched against BacMet (v 2.0, with biocides resistance genes discarded) [11] by DIAMOND using identical parameters. Besides, enrichM (v.0.4.0, https://github.com/geronimp/enrichM), interproscan (v5.39-77.0, https://github.com/ebi-pf-team/interproscan), and eggnog-mapper [49] (v1.0.3) were used to perform a more comprehensive annotation against Kyoto Encyclopedia of Genes and Genomes (KEGG) [50], Gene Ontology, and EggNOG (v5.0) database [51], respectively.

### Enrichment analysis

We performed enrichment analysis using GSEA [52] (v.4.0.2) based on protein expression differences between the heavy metal-treated groups and the control group (see Additional file 1: Text S7 for details). Enrichment network was created by *EnrichmentMap* package in Cytoscape [53] (v3.7.2) for ontologies with normalized enrichment score > 1, *p*-value < 0.05, and false discovery rate (FDR) < 0.25 (to recover more differentially enriched gene sets, a more lenient FDR threshold up to 0.25 can be used [54]), and ontologies were linked and clustered with shared edges using the similarity cutoff > 0.25.

The leading-edge subset of each enriched gene set was obtained using default settings. Genes in the subset contributed the most to the enrichment of corresponding functionality. Here, microbes that expressed (i.e., identified by metaproteomics) gene(s) in the subset were defined as core-functioning microbes, representing key microorganisms contributing the most to the enrichment of specific functions.

### MAG co-occurrence network

The co-occurrence network of MAGs (with average abundance > 1 CoPM and occurrence at least in two-thirds of the samples) was constructed by CoNet (v2) using an ensemble approach and the ReBoot technique [55] (Additional file 1: Text S8). Only edges with FDR < 0.05 were reserved for downstream analysis. Edge assigned as “co-presence” and “mutual exclusion” indicate positive (e.g., cooperative) and negative (e.g., competitive) relationship between a pair of MAGs, respectively. Topology features (i.e., degree and betweenness centrality) for each node were calculated using R [56] package *igraph* [57].

### Statistical analysis

The abundance of MAG in each sample was indicated by the number of genome copies per million reads (CoPM) calculated by the *quant_bins* module of MetaWRAP. The percentage of the total community captured by MAGs was estimated based on the recovery of ribosomal protein genes using singleM v0.13.0 as described [58]. Linear discriminant analysis (LDA) was performed using LEfSe [59] to find significantly more abundant MAGs, whose LDA score > 2 (*α*=0.05) in each reactor. The label-free quantified raw abundance of protein was normalized by the genetic abundance (in CoPM) of corresponding MAG. As for the protein with alignments in multiple MAGs, its abundance was normalized using the summed-up abundance of the corresponding MAGs. Then, the fold changes of both the raw abundance and normalized abundance of proteins in the heavy metal-treated group compared to that in the control group were calculated using *DESeq*2 [60] (v.1.22.2). Only proteins with independent hypothesis weighted [61] Benjamini and Hochberg FDR adjusted *p* < 0.05 and log_2_(fold change) > 1 or < −1 were regarded as significantly “up-expressed” or “down-expressed”. Variances of nutrient removal rate, bacterial viability, and SOUR between heavy metal-treated reactors and the control reactor were assessed using Student’s *t*-tests with *ggpubr* R package. Meanwhile, independent group Welch’s equivalence test was performed to determine whether the reactors’ nutrient removal rates were equivalent using *TOSTER* R package [62].

## Results

### Functional microbes inhabiting DPRS

**Fig. 1:**
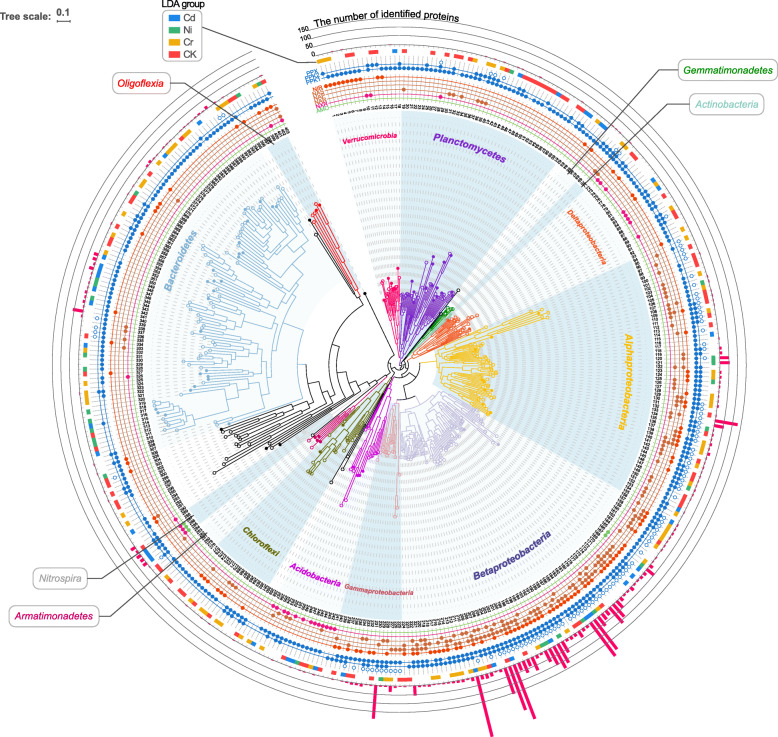
Phylogenetic tree of medium- and high-quality metagenome-assembled genomes (MAGs). Dots embedded in the circles outside the tree indicate the presence of functional genes (AMO, ammonia monooxygenase; NXR, nitrite oxidoreductase; NAR, membrane-bound nitrate reductase; NAP, periplasmic dissimilatory nitrate reductases; NAS, assimilatory nitrate reductase; NIR, nitrite reductase; PPK, polyphosphate kinase; and PPX, exopolyphosphatase) in the corresponding MAG. The outer strip indicates the significantly more abundant MAGs whose linear discriminant analysis (LDA) scores > 2 (α=0.05), with colors referring to the reactors they belong to. The outer bar graph indicates the number of metaproteomics-identified protein(s) in each MAG

A total of 403 MAGs that met with the quality criteria were recovered, capturing 47.1 ± 5.8% of the identified ribosomal protein genes in DPRS metagenomes (Additional file 1: Fig. S1). By comparing the taxonomic profile of recovered MAGs (Fig. 1 and Additional file 2) with the taxonomic profile predicted from ribosomal protein gene *rplB* in metagenomes (Additional file 1: Fig. S1), the recovered MAGs were confirmed to be the representative of the dominated taxa in DPRS.

Dissimilatory nitrate reductases (membrane-bound NAR and periplasmic NAP), assimilatory nitrate reductases (NAS), and nitrite reductases (NIR), the gene markers of denitrifiers [46], were identified in 114 MAGs, 58 MAGs, and 116 MAGs, respectively (Fig. 1 and Additional file 1: Fig. S2). Likewise, nitrite oxidoreductase (NXR), the indicator for nitrite oxidizers, was another widespread trait, with 31 MAGs found to encode a homolog (Additional file 1: Fig. S3). Conversely, the ammonia monooxygenase (i.e., AMO) gene was conserved in genera *Nitrosomonas* (MAG-166, 167) and *Nitrospira* (MAG-285~287). Thereinto, three *Nitrospira* spp. were also putative nitrite oxidizers, suggesting their potential to perform complete ammonia oxidation to nitrate (comammox).

The trait for polyP synthesis using polyphosphate kinase (PPK) was dispersed in over 89% of the MAGs. But actually, only phosphorus-accumulating organisms (PAOs) can effectively exhibit this highly specialized phenotype [63]. Therefore, only the well-known PAO *Candidatus* Accumulibacter (7 MAGs) and putative PAO *Dechloromonas* (5 MAGs) [47] were potentially responsible for the phosphorus removal. The presence of PPK (*ppk1* and *ppk2*) and exopolyphosphatase in these PAOs’ genomes ensure their active roles in the synthesis and degradation cycle of polyP metabolism. Besides, these PAOs were all potential denitrifiers except for MAG-190, showing versatility in nutrient removal. Although *Ca.* Accumulibacter and *Dechloromonas* were two different genera, they shared > 70% genomic similarity (based on the average nucleotide identity and amino acid identity, Additional file 1: Fig. S4), implying their close relationship in phylogeny and high similarity in functional potentials.

### Thousands of proteins were detected and quantified by metaproteomics

We obtained 9537 unique proteins, of which 7973 had valid label-free quantification, from the DPRS metaproteomes at the high-pressure stages. The protein expressions were stable within each reactor across the sampling periods, but only 3341 (35%) proteins were constantly detected in all reactors (Additional file 1: Fig. S5). This indicated the distinct metaproteome compositions caused by heavy metals, which was also indicated by the Bray-Curtis distance-based principal component analysis.

By mapping metaproteomes back to MAGs, 2652 proteins were uniquely assigned to specific MAGs, while 625 proteins had multiple alignments (Fig. 1). Most of these proteins were affiliated with MAGs belonging to *Betaproteobacteria*, with the highest number found in *Ca.* Accumulibacter (1008), followed by *Dechloromonas* (571). The high recovery rates of proteins in PAOs might suggest their intensive activities under heavy metal pressure. Besides, other taxa such as *Zoogloea* (513) and *Comamonas* (357) also had numerous proteins detected.

### Nitrifiers determined the removal of nitrogen under heavy metal-constrained oxygen availability

**Fig. 2:**
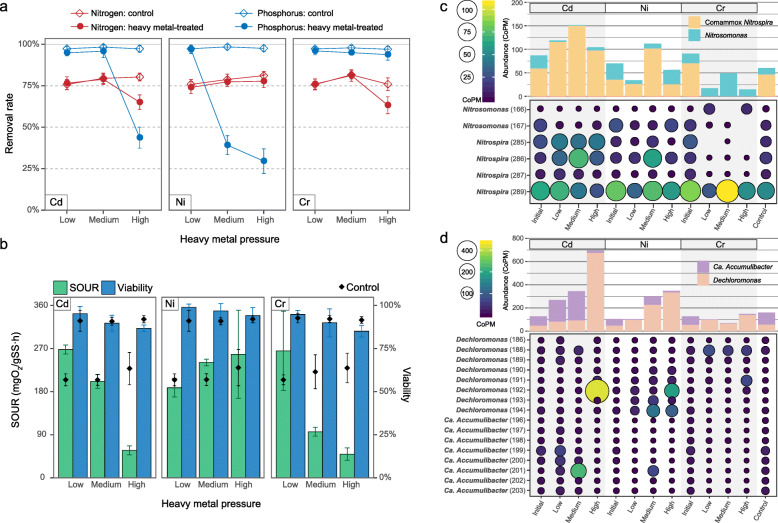
Maintenance of reactors’ performance and functional microbes under heavy metal pressure. **a** The removal rates of nitrogen and phosphorus during stable phases (see additional file 1: Fig. S6). **b** Overall microbial viability and specific oxygen uptake rate (SOUR). Error bars indicate the standard deviation of corresponding indices estimated from at least nine replicates. **c** Abundance of nitrifiers *Nitrospira* and *Nitrosomonas*. **d** Abundance of phosphorus-accumulating organisms (PAOs) *Candidatus* Accumulibacter and *Dechloromonas*. The abundance of individual species is indicated by the coverage of the corresponding metagenome-assembled genome (MAG) in copies of genome per million reads (CoPM). Stacked bar summarizes the total abundance of the specific taxon

After the initial perturbations at the low-pressure and medium-pressure stages, total nitrogen removal rates in heavy metal-treated reactors remained equivalent to that in the control (Welch’s equivalence test’s *p* < 0.05, Fig. 2a and Additional file 1: Fig. S6 and Table S5). But the removal rate dropped significantly by 10% and 13% at the high-pressure stage under Cd pressure (*t*-test’s *p*=6.0 ×10^−13^) and Cr pressure (*t*-test’s *p*=7.2 ×10^−12^), respectively. In contrast, Ni exhibited a minor impact on nitrogen removal performance even at the high-pressure stage. During the unfavorable performance under Cd and Cr exposure, SOUR decreased by 80%, while microbial viability exhibited relatively minor reduction (< 7%, Fig. 2b). In conjunction, ammonia became the primary speciation of residual nitrogen in the effluent (Additional file 1: Fig. S6). These phenomena suggested an inefficiency of oxygen utilization caused by high-concentration Cd or Cr, which made nitrification the limiting step of nitrogen removal.

**Fig. 3:**
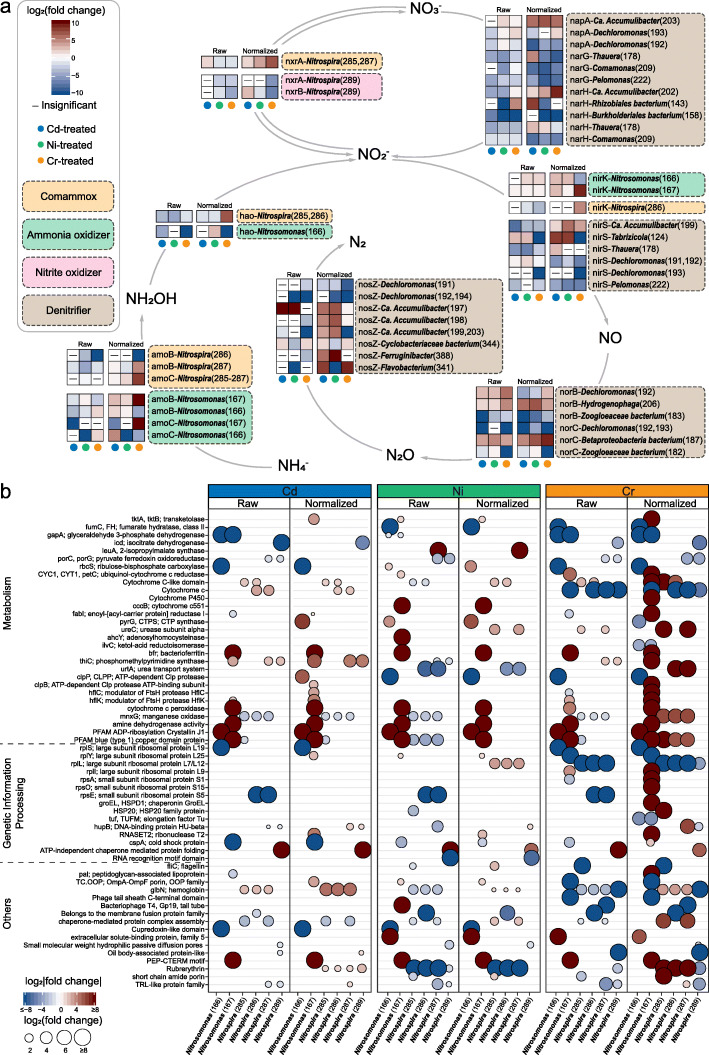
Functional changes at the high-pressure stages associating with nitrifiers. **a** Functional changes of nitrifiers and their roles in nitrification and denitrification. **b** Differentially expressed proteins (excluding those in subfigure **a**) by *Nitrospira* (both the comammox spp. and the specialized nitrite oxidizer sp.) and *Nitrosomonas*. The fold change of protein expression is calculated based on its raw/normalized abundance in the heavy metal-treated group compared with that in the control group. Only these with adjusted *p*-value < 0.05 and log2 fold change > 1 or < −1 are shown

Oxidizing ammonia to nitrite, the first step of nitrification, was exclusively conducted by *Nitrosomonas* and comammox *Nitrospira* (Fig. 3a). However, those two taxa distinctively responded when treated with heavy metals. Generally, the comammox *Nitrospira* exhibited a greater survival advantage under Cd pressure, with all the comammox spp. showing significantly (LDA score > 3) higher abundance than in other conditions (Figs. 1 and 2c). They oxidized ammonia efficiently (>99%) despite the presence of low- or medium-concentration Cd (Additional file 1: Fig. S6). Even when oxygen availability was largely constrained by high-concentration Cd, 83 ± 10% of ammonia was still successfully transformed. The overproduction of highly oxygen-affinitive hemoglobin (i.e., *glbN*) and electron-transporting cytochrome c-like proteins by comammox *Nitrospira* spp. (Fig. 3b) had implications for their competitive advantages in oxygen acquisition and utilization. Conversely, *Nitrosomonas* dominated over the comammox *Nitrospira* in survival under Cr pressure (Fig. 2c). In particular, *Nitrosomonas* sp. MAG-166 enhanced proliferation and overexpressed AMO at the high-pressure stage (Fig. 3b). Eventually, Cr-treated DPRS maintained an ammonia removal rate of 73 ± 6% at the high-pressure stage. While under Ni pressure, the abundance of both *Nitrospira* spp. and *Nitrosomonas* spp. kept relatively stable (Fig. 2c). Also, the ammonia removal performance was left intact even at the high-pressure stage, despite the overall inhibited expression of AMO. Therefore, the decrease in oxygen availability rather than the decline in AMO expression was more prone to inhibit ammonia oxidization.

Although potential nitrite oxidizers were taxonomically diverse in DPRS, only *Nitrospira* (including the nitrite-oxidizing specialized sp. MAG-289) expressed NXRs to a detectable level, indicating *Nitrospira* was probably the most active nitrite oxidizers in DPRS. Under high-concentration Cd exposure, *Nitrospira* slightly overproduced NXRs. Meanwhile, denitrifiers collectively attenuated denitrifying activities (Fig. 3a). However, given the nitrite accumulation and nitrate consumption (Additional file 1: Fig. S6), NXRs expressed by *Nitrospira* may promote nitrate reduction rather than nitrite oxidization under limited oxygen availability. Nitrite was then reduced to nitric oxide by NIR producers such as *Nitrosomonas* sp. MAG-167 (Fig. 3a). A similar phenomenon was observed when the NXR expression of *Nitrospira* was inhibited under Cr pressure. Nitrite cannot be oxidized to nitrate and therefore accumulated (Additional file 1: Fig. S6). This stimulated *Nitrosomonas* (sp. MAG-167), *Pseudoxanthomonas*, and *Dechloromonas* to overexpress NIR for nitrite reduction (Fig. 3a). In short, nitrifiers tended to perform partial nitrification and denitrification process for nitrogen removal when oxygen availability was constrained.

### *Dechloromonas* outcompeted *Ca.* Accumulibacter under heavy metal pressure

Phosphorus removal in DPRS was determined by PAOs *Ca.* Accumulibacter and *Dechloromonas* (Fig. 1). *Ca.* Accumulibacter sp. MAG-201 showed significantly (LDA score > 4) higher abundance under Cd pressure. However, its survival advantage evened out at the high-pressure stage. Instead, *Dechloromonas* spp. became more abundant and contributed to the dominance of PAOs in DPRS (Fig. 2d). The survival advantage of *Dechloromonas* was also observed under Ni pressure. Comparatively, Cr exhibited suppressive effects on both *Ca.* Accumulibacter and *Dechloromonas*. Nevertheless, *Dechloromonas* was still more abundant than *Ca.* Accumulibacter.

**Fig. 4:**
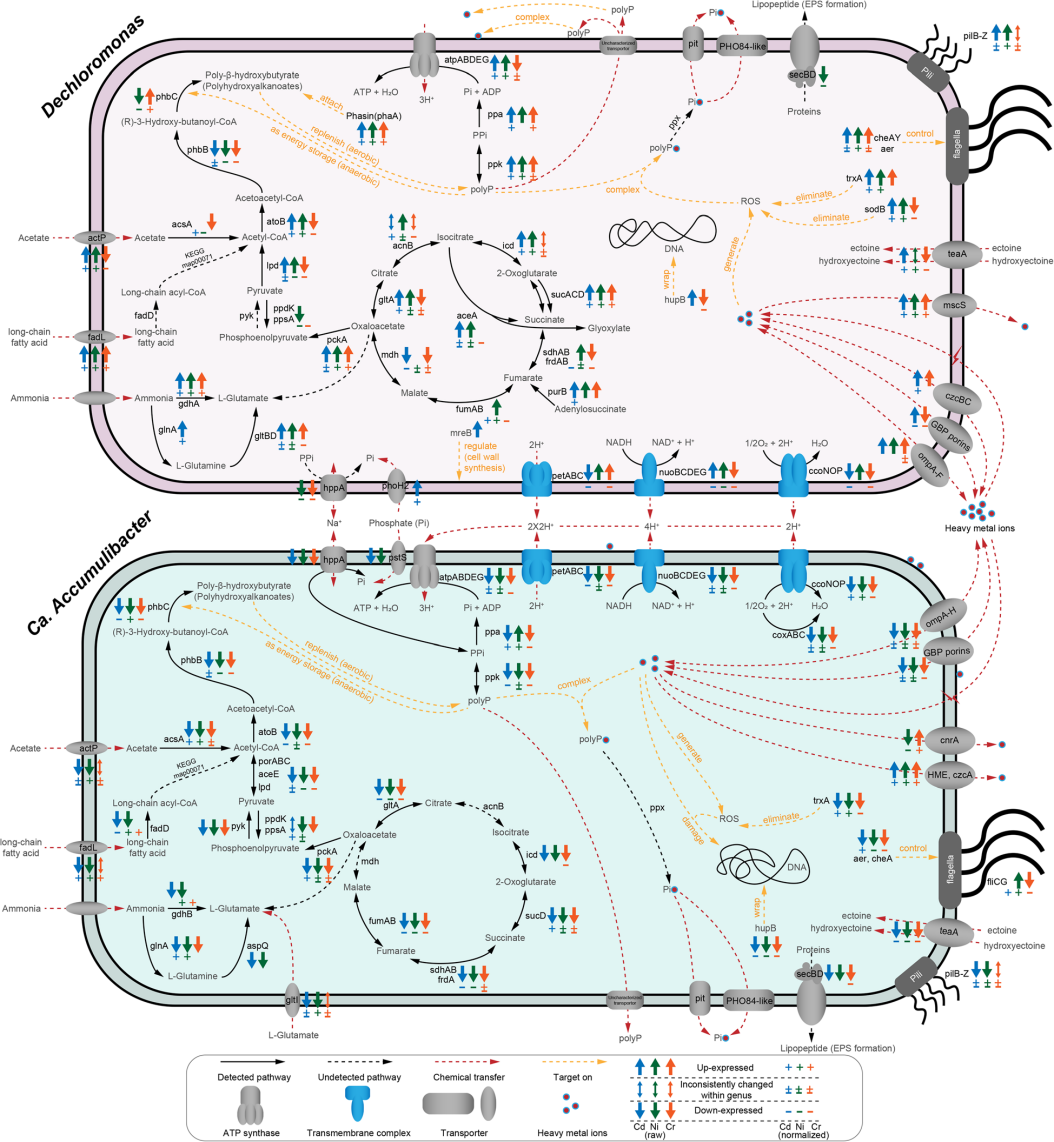
Comparisons of functional shifts at the high-pressure stages between *Dechloromonas* and *Candidatus* Accumulibacter. Only these significantly changed (adjusted *p*-value < 0.05) proteins involving energy metabolism or heavy metal resistance are shown

Generally, proteins in over 898 protein families were variedly expressed in these PAOs (see Additional file 3). Most of the proteins associated with *Ca.* Accumulibacter were downexpressed at the high-pressure stages, which was in line with its decreased abundance. Comparatively, *Dechloromonas* had more proteins upexpressed, especially under Cd and Ni pressure (Fig. 4 and see Additional file 1: Fig. S7 for the genome-specific profile). For instance, the expression of PPK in *Ca.* Accumulibacter spp. was substantially mitigated while that in *Dechloromonas* spp. was increased up to 23 folds and 30 folds after being treated by high-concentration Cd and Ni, respectively. The overexpression of PPK by *Dechloromonas* suggested that high-concentration heavy metals stimulated its polyP synthesis activity. Also, the reduction in polyhydroxyalkanoates synthesis (i.e., acetoacetyl-CoA reductase *phbB* and poly(3-hydroxyalkanoate) polymerase *phbC*) had implications for the decreased consumption of polyP. Besides, *Dechloromonas* also outcompeted *Ca.* Accumulibacter concerning energy utilization (e.g., TCA cycle, oxidative phosphorylation, and fatty acids utilization), nitrogen assimilation (e.g., glutamine synthetase *glnA*), peroxide elimination (i.e., thioredoxin *trxA* and superoxide dismutase *sodB*), and stress response activities (e.g., DNA-binding *hupB* and chemotaxis *cheA*).

Interestingly, excessive orthophosphate (approximately 2 mg/L) was observed in the effluent under high-concentration Cd or Ni exposure but not Cr pressure (Additional file 1: Fig. S6). Given the higher abundance of PAOs and polyP synthesis activity under Cd and Ni pressure (Fig. 4), the excessive orthophosphate was probably in the form of inorganic polymer (i.e., polyP) released by PAOs [64] or from polyP hydrolysis [15]. To confirm this speculation, we investigated the phosphorus removal and polyP-accumulating performance of heavy metal-treated DPRS after removing heavy metal pressure (Additional file 1: Fig. S8). As expected, phosphorus removal performance recovered immediately, and PAOs significantly (*t*-test, *p* < 0.01) increased polyP accumulation. Such phenomena underpinned our speculation that more polyP was used for heavy metal detoxification than as energy storage under heavy metal pressure. Besides, when polyP accumulated extracellularly, considerable reductions in the removal rate of Cd and Ni were observed (Additional file 1: Fig. S6), which was consistent with the abatement of intracellular accumulation of Cd and Ni (Additional file 1: Fig. S9). Therefore, polyP complexation was a potential mechanism contributing to the resistance for Cd and Ni in particular.

### Shifts in functional profiles indicated the most active mechanisms for heavy metal resistance

**Fig. 5:**
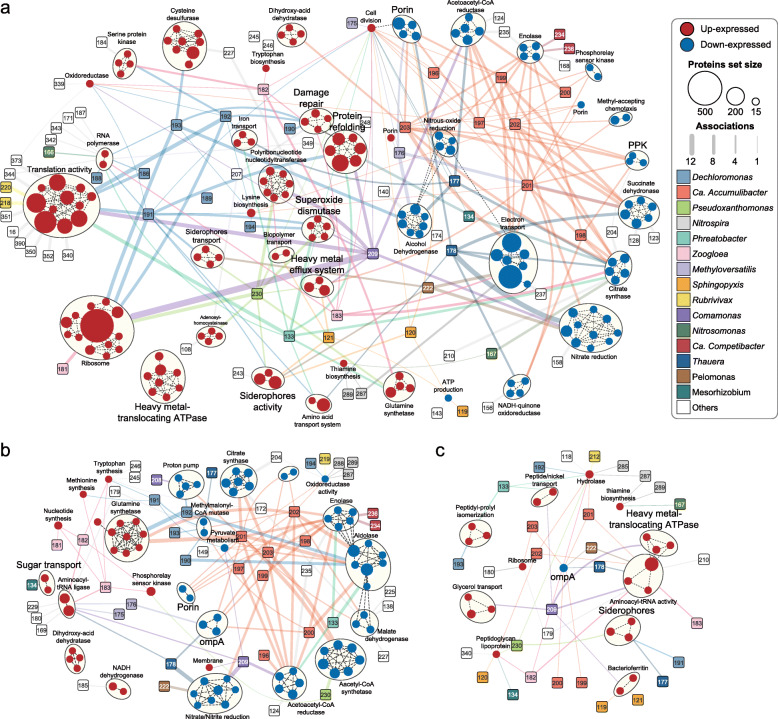
Enriched functions and associating core functioning microbes at the high-pressure stages. Circles refer to significantly upexpressed (red) and downexpressed (blue) ontologies in heavy metal-treated groups estimated by GSEA. Those with Jaccard combined overlap > 0.25 are automatically clustered (by dashed lines) and summarized using Cytoscape application clusterMaker2 and WordCloud, respectively. Rounded squares indicate the core-functioning microbes that contributed the most to the changes of their linked (by solid lines) functions. The number inside the square is the ID number of the corresponding metagenome-assembled genome (MAG). The solid line’s width reflects the number of associations between the microbe and the ontologies within the cluster. For clarity, only associations > half of the number of ontologies in each cluster are shown (full data is available in Additional file 4). **a** Cd treated. **b** Ni treated. **c** Cr treated

To get a more comprehensive view of the most responsive heavy metal-resistance mechanisms, we characterized the differentially expressed proteins at the high-pressure stages using enrichment analysis. Generally, the analysis discriminated 142 significantly upexpressed and 94 significantly downexpressed protein ontologies (representing specific functions) in heavy metal-treated DPRS. The core functioning microbes contributing to these significantly enriched functions were also identified. *Nitrospira*, *Nitrosomonas*, *Dechloromonas*, and *Ca.* Accumulibacter were among the most responsive taxa, for they consistently contributed to the functional changes of the community regardless of the metal speciation (Fig. 5).

The functional profile under Cd pressure changed the most, with 107 ontologies positively enriched while 66 ontologies negatively enriched. Protein synthesis was extensively promoted, with various taxa (44 MAGs), including *Dechloromonas*, *Sphingopyxis*, and *Pseudoxanthomonas* overexpressing ribosomal proteins (e.g., *rpsB* and *rplF*) and translation-relevant proteins (e.g., elongation factor *fusA* and *tuf*). These genera contributed the most to the positively enriched functions (Additional file 1: Fig. S10), including RNA polymerase (e.g., *rpoA*), amino acid synthesis (e.g., *livK*), and cell division (e.g., *ftsA*) activities, which in turn facilitated their growth and dominance under Cd pressure (Fig. 2, Additional file 1: Figs. S2 and S3). *Dechloromonas* spp., *Phreatobacter* sp., and *Comamonas* sp. also overproduced chaperonin *groEL*, superoxide dismutase (e.g., *sodA*), and heat response proteins (e.g., *dnaGK*) to relieve oxidative stress and facilitate the repairing of damaged cell components. Moreover, *Pseudoxanthomonas* sp., along with *Sphingopyxis* sp. and *Pelomonas* sp., was stimulated to enhance siderophore-associating activity (e.g., *fiu*). Meanwhile, increased heavy metal exporting ATPase (e.g., *pbrA* and *czcP*) and efflux (e.g., *czcABC*) activities were observed in *Pseudoxanthomonas* sp. and *Sphingopyxis* sp., respectively. Comparatively, mitigated functions were mainly associated with *Ca.* Accumulibacter. For example, the reduction of outer membrane porins (both raw and normalized abundance) may relate to its decreased cellular permeability*.* Furthermore, degraded enolase (phosphopyruvate hydratase) related to *Ca.* Accumulibacter and *Candidatus* Competibacter had implications for the enhanced protein-exporting activity [65]. This was accordant with the higher concentration of proteins in EPS under Cd pressure than that in the control (Additional file 1: Fig. S11).

A moderate change in functional profile was observed in Ni-treated DPRS, with 24 ontologies positively enriched, while 57 ontologies negatively enriched (Fig. 5b). The overall functional profile in Ni-treated DPRS shared a higher similarity with that in Cd-treated DPRS than with those in other conditions (Additional file 1: Fig. S5). For example, the degraded enolase expression of *Ca.* Accumulibacter and *Ca.* Competibacter was concordantly observed in Ni-treated and Cd-treated DPRS. This explained the overproduction of extracellular proteins under Cd and Ni exposure (Additional file 1: Fig. S11), which could contribute to the entrapment of heavy metals (Additional file 1: Fig. S12). Nonetheless, several proteins involved in heavy metal-resistance varied differently in Ni-treated DPRS and Cd-treated DPRS. For instance, the overexpression of ABC-type sugar-transporting proteins was only found under Ni exposure, which may be related to the higher production of extracellular polysaccharides (Additional file 1: Fig. S11). Interestingly, although siderophore-producing *Sphingopyxis* was abundant, no significant enhancement on the siderophore activity was found.

In the Cr-treated DPRS, differentially expressed proteins were scattered in functionality as only 23 ontologies significantly varied (Fig. 5c). Nevertheless, several mechanisms having potentials for heavy metal resistance were significantly enriched. For instance, the increased expression of peptidoglycan-associated lipoprotein (i.e., *pal*) by several taxa such as *Pseudoxanthomonas*, *Sphingopyxis*, and *Flavobacterium* implied their reinforced membrane integrity. Like the situations under Cd stress, *Pseudoxanthomonas* sp., *Acinetobacter* sp., and *Sphingopyxis* sp. increased siderophore production, and meanwhile, *Pseudoxanthomonas* sp. also facilitated heavy metal-exporting activity. Notably, the reduction of outer membrane porins was found continuously in all heavy metal-treated groups and the only negatively enriched function under Cr pressure.

### Estimated interspecific interactions revealed potential altruistic mechanisms for the community-wide resistance

**Fig. 6:**
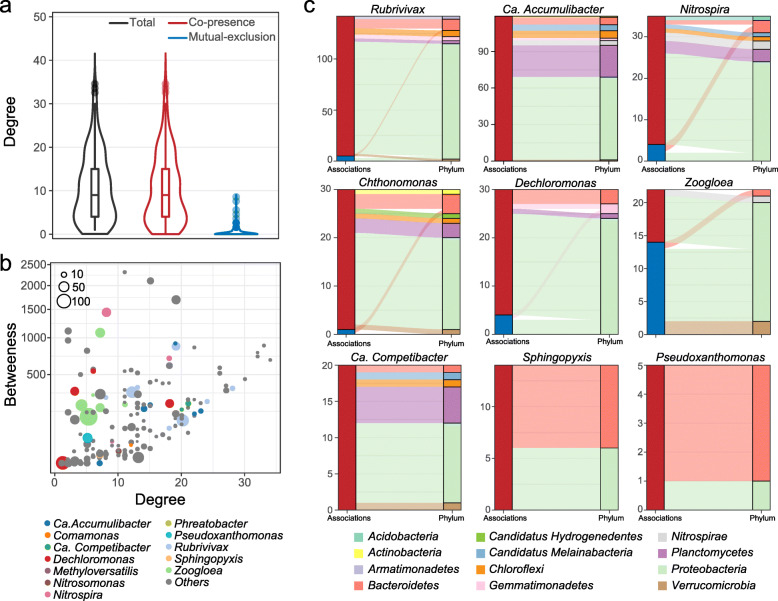
Interspecific interactions estimated from the co-occurrence network. **a** Distributions of degree in co-occurrence networks with all associations, only co-presence associations, and only mutual-exclusion associations. **b** Degree and betweenness centrality of microbes in co-occurrence network. Node color and size indicate taxonomy and the average abundance of metagenome-assembled genome (MAG), respectively. **c** Sankey diagrams exhibit the relationships of specific taxa with other microbes. Left column shows the number of relationships associating with that taxon, and the colors of it indicate the relationship type (red for co-presence relationship while blue for mutual-exclusive relationship). Colors of the right column and alluvium indicate the taxonomy of their related microbes

Microbe-derived substances involving the extracellular detoxification of heavy metals could benefit not only the producers but also other members of the community. In addition, some taxa (e.g., *Nitrospira*) that were not supposed to resist heavy metals were indeed found abundant under heavy metal stresses. To characterize such potential public good, we generated a MAG co-occurrence network based on the estimated interspecific associations (Additional file 1: Fig. S13). The network consisted of 181 microbes (as nodes) and 964 interspecific associations (as edges). Generally, the DPRS microbiome prefers cooperative interactions than competitive relationships under heavy metal pressure, with most of the estimated associations being positive (i.e., co-presence, Fig. 6a) while only 33 interactions being negative (i.e., mutual-exclusion).

Species belonging to *Rubrivivax*, *Ca.* Accumulibacter, *Nitrospira*, *Chthonomonas*, *Dechloromonas*, *Zoogloea*, and *Ca.* Competibacter shared numerous interactions with other taxa. In particular, *Ca.* Accumulibacter spp., *Dechloromonas* sp., *Ca.* Competibacter spp., and *Rubrivivax* spp. had a high degree (≥ 15) but low betweenness (≤ 250) centralities (Fig. 6b and Additional file 1: Fig. S13), located in the keystone positions of the network [66]. Given the high proportion of positive associations relating to PAOs (both *Ca.* Accumulibacter and *Dechloromonas*), polyP could be a public good that benefited various taxa, including other phyla (Fig. 6c). The potential EPS producer, *Ca.* Competibacter also exhibited co-presence patterns with many other taxa, indicating that its presence was vital for its adjacent members in the network. Comparatively, siderophore-producing *Pseudoxanthomonas* and *Sphingopyxis* were more peripheral in the network. Nevertheless, several taxa such as *Cyclobacteriaceae* and *Chitinophagaceae* were in favor of their presence.

## Discussion

Heavy metal contamination is a common factor that affects built ecosystems in WWTPs [1, 3, 8, 9]. Studying the response of these ecosystems and microbiomes therein during heavy metal exposure can elucidate key features involved in microbial adaptation to toxicants and improve the ecosystem robustness for wastewater treatment. However, the functional changes and heavy metal-resistance strategies of individual microorganisms in DPRS remain poorly understood. Here, we studied the adaptive process of the DPRS microbiome under different heavy metal stresses using genome-centric metaproteomics. It showed that microbes adjusted their nutrient metabolisms to suit the heavy metal-constrained conditions and therefore maintain their viability. Furthermore, several resistance mechanisms associated with different microbes were revealed, which reflected the functional diversity and heterogeneity of the DPRS microbiome in heavy metal resistance. To our knowledge, this is the first time the heavy metal-resistance mechanisms in this complex microbial community were characterized using genome-centric metaproteomics.

We characterized the strategies used by nitrifiers (*Nitrospira* and *Nitrosomonas*) to maintain nitrification under heavy metal-constrained oxygen availability. Constrained oxygen utilization (indicated by decreased SOUR, see Fig. 2b) is a common restriction resulting in attenuated nitrification activity in heavy metal-contaminated sludge systems [9]. We found that comammox *Nitrospira* dominated over *Nitrosomonas* in Cd-constrained conditions (Fig. 2c). Interestingly, such predominance of comammox *Nitrospira* was recently reported in nitrifying reactors [67] and a full-scale WWTP [68] operated under constant anoxic conditions. The advantage of *Nitrospira* in anoxic conditions indicates its outstanding ability for oxygen acquirement [69]. Three species of comammox *Nitrospira* significantly overproduced (up to 4.4 fold) hemoglobin (Fig. 3b), which is known to facilitate oxygen delivery to oxygen-utilizing enzymes in bacteria [70], and pivotal to the survival and adaptation of aerobic bacteria under hypoxia [71]. Comammox *Nitrospira* probably overproduced hemoglobin to maintain oxygen supply for ammonia oxidization when oxygen availability was constrained. Besides, overproduced cytochrome c-like proteins may compensate for the Cd-caused inhibition on the respiratory electron transport chain involved in nitrogen metabolism [72]. Unlike comammox *Nitrospira*, *Nitrosomonas* dominated under high-concentration Cr pressure and overproduced AMO under additional constrained oxygen availability (Figs. 2c and 3a). Similarly, the AMO transcriptions were observed increased in *Nitrosomonas europaea* pure cultures during oxygen-limited growth [73] and a nitrifying enrichment culture when its SOUR was constrained by heavy metals [9]. Overproduced AMO may contribute to the maintenance of ammonia oxidization under Cr pressure [74]. Normally, a partial nitrification and denitrification process are achieved when the growth of nitrite oxidizers is inhibited [46], which is the case under high Cr pressure. However, comammox *Nitrospira* also oxidized ammonia to nitrite despite the overproduction of NXRs under Cd exposure. This phenomenon supports a recent hypothesis that comammox bacteria act as specialized ammonia oxidizers in oxygen-limited systems [46]. Furthermore, both the accumulation of nitrite and the consumption of nitrate were observed (Additional file 1: Fig. S5), though denitrifiers collectively decreased the expressions of genes involved in nitrate and nitrate reduction (Fig. 3a). In conjunction with the fact that NXR has the experimentally proven ability to catalyze nitrate reduction [75], we speculate that *Nitrospira*-like NXRs tend to catalyze nitrate reduction rather than nitrite oxidization under constrained oxygen availability. Nonetheless, NXRs could also be simply inactivated by limited oxygen availability because the expression of an enzyme does not necessarily equal its activity [76]. Given the importance of NXRs in nitrite and nitrate metabolism, further experiment concerning this enzyme’s activity under hypoxia is warranted.

As for PAOs, we found that *Dechloromonas* was more abundant than the canonical PAO *Ca.* Accumulibacter under heavy metal pressures (Fig. 2d). Only very recently, a *Dechloromonas* sp. was shown to accumulate high levels of polyP and proposed as a potential PAO [77]. Here, we provided proteomic evidence for PPKs associated to *Dechloromonas* spp. for the first time, further confirming their active roles as PAOs. In addition, given their increased abundances and expressions of PPKs (Figs. 2 and 4), *Dechloromonas* spp. may even contribute to the enhanced polyP synthesis under high-concentration Cd or Ni exposure (Additional file 1: Fig. S8). Previous 16S rRNA gene-based studies have reported similar increases in *Dechloromonas*’s abundance after treatment by polyaluminium chloride [78], cadmium [8], and copper [79] in sludge systems, indicating its remarkable resistance to metallic toxicants. Conversely, *Ca.* Accumulibacter spp. were susceptible to high-concentration heavy metals, probably because they can hardly export polyP or polyP-heavy metal complexes extracellularly for detoxification as efficient as *Dechloromonas* did (Fig. 4). Besides, the compositions of polyP produced by *Ca.* Accumulibacter and *Dechloromonas* are distinct, which may also result in their different affinities in chelating heavy metals [80]. PolyP has proved essential to the heavy metal resistance of various microorganisms [64]. However, its protective role in complex microbial communities was scarcely reported [21]. This is probably because previously investigated communities were those lacking dynamic dissolved oxygen fluctuations such as soils [25], sediments [24], and activated sludge [81]. Without dynamic dissolved oxygen fluctuations, PAOs can hardly outcompete other microbes nor effectively perform polyP synthesis [47]. Here, we propose that polyP can contribute to the heavy metal resistance of complex microbial communities when PAOs (especially *Dechloromonas*) are dominated. Moreover, this trait not only protected PAOs themselves but also had the potential to benefit other members in the community during heavy metal resistance (Fig. 6c). However, polyP’s merit as a public good was mainly speculated based on the estimated interspecific associations. Further experimental confirmation of the direct interactions between polyP and heavy metals in complex microbial systems is required.

Besides polyP synthesis, functional responses to heavy metals were largely different between *Dechloromonas* spp. and *Ca.* Accumulibacter spp., despite their high genomic similarities. *Ca.* Accumulibacter spp. consistently reduced outer membrane permeability under heavy metal stresses (Figs. 4 and 5), which can potentially mitigate the passive uptake of heavy metals [21]. However, this mechanism gave *Ca.* Accumulibacter spp. no advantage in survival (Fig. 2), probably because the reduced permeability also attenuated the uptake for nutrients and other essential substances [18]. In contrast, *Dechloromonas* spp. increased *ompA*-like porin expression. Additionally, they potentially enhanced fatty acid uptake and utilization (Fig. 4), which could result in a substrate-depleted environment for *Ca.* Accumulibacter. Therefore, reducing porins may be unsuitable for niche conservation when interspecific competition for substrate exists. Furthermore, *Dechloromonas* overproduced chaperonin, superoxide dismutase, DNA-damage repairing, and stress response proteins (Figs. 4 and 5), which were known to relieve damages caused by reactive oxygen species [82]. In short, comparisons between *Ca.* Accumulibacter spp. and *Dechloromonas* spp. demonstrate that differences in heavy metal resistance can overturn the competition hierarchy between members with niche overlap.

We also identified several actively functioning mechanisms associated with other taxa in virtue of the enrichment analysis. For example, microbes belonging to *Ca.* Accumulibacter, *Ca.* Competibacter, and *Mesorhizobium* can contribute to the EPS overproduction under heavy metal stresses. The overproduction of proteins and polysaccharides was consistent with the considerable amounts of Cd and Ni detected in EPS (Additional file 1: Figs. S11 and S12). The reason for this may be soft acid (Cd) and borderline acid (Ni) can associate tightly with proteins and polysaccharides via soft bases such as thiols, according to Pearson’s acid base theory [83]. Besides, polyP can also accumulate in EPS and amend the adsorption capacity of EPS [15, 84]. Consequently, EPS as the main component of sludge flocs was likely an ideal barrier to trap Cd and Ni and worked as an important detoxification strategy. Moreover, EPS may protect not only its producers but also coexistent microbes in their vicinity (Fig. 6c). However, EPS failed to capture highly redox-active Cr probably because Cr tended to decompose EPS (Additional file 1: Figure S11) rather than bind with it [10], indicating that the entrapment by EPS was not a panacea for all heavy metals.

Another example is siderophores, the iron-affinitive chelators secreted by siderophore-producing microbes [85]. Besides iron, siderophores can chelate heavy metals and block their entrance into microbial cells [17]. In DPRS, *Pseudoxanthomonas* sp. and *Sphingopyxis* spp. were identified as the most active siderophore-producing microbes. They contributed the most to the overexpression of siderophore-related functions under Cd and Cr pressure (Fig. 5). However, *Sphingopyxis* sp. had no siderophore-related proteins overproduced despite its dominance under Ni pressure. This is possibly because Ni is one of the few heavy metals that can accumulate in cells through the siderophore-mediated transport system [86]. Overproducing siderophores would not give the producers advantages in resisting Ni. The inconsistency in functional responses indicated microorganisms could change their phenotype in heavy metal resistance based on heavy metals’ properties. Furthermore, *Pseudoxanthomonas* and *Sphingopyxis* overexpressed MRGs, including heavy metal efflux system components (e.g., *czcABC*) and heavy metal translocating P-type ATPase (e.g., *copA*), to export heavy metals. Consequently, siderophore-producing bacteria can benefit from both siderophores and the heavy metal efflux system.

Due to the limitation of genome-binning approaches and the high complexity of the DPRS system [87], some genes were not binned into any MAG. Therefore, it was difficult to assign a precise taxonomy to some differentially expressed proteins. Microbes without recovered MAGs should also contribute to the heavy metal resistance but cannot be characterized extensively in this study. Future advances in sequencing technologies and metagenomic binning algorithms may break through these limitations. Nevertheless, the MAGs recovered in this study were soundly representative of the dominant taxa (accounted for 63.4 ± 4.9% of reads in metagenomes, see Additional file 1: Fig. S1) in DPRS, and these identified core functioning microbes provided informative knowledge of the active taxa in heavy metal resistance. Besides, our focus on nutrient-removing microbes has practical value for optimizing DPRS’s robustness in nitrogen and phosphorus removal.

## Conclusions

Our work investigated the adaptative and resistive features of DPRS microbiomes under heavy metal pressure in a genome-scale resolution. Results demonstrated that microbes adopted taxon-specific strategies to resist toxic heavy metals and therefore contributed to the robustness of functional diversity within the community. *Nitrospira* enhanced oxygen utilization while *Nitrosomonas* overexpressed related enzymes to maintain partial nitrification under heavy metal-constrained conditions. Our study also highlights the outstanding resistance of PAO *Dechloromonas* under heavy metal pressure and the potential of polyP in heavy metal detoxification for the whole community. Other functions, including EPS formation, siderophore production, superoxide elimination, heavy metal efflux, and damage repairing, also significantly contributed to the heavy metal resistance of various taxa in DPRS. These mechanisms and relevant microbes suggest the emphases for future investigations concerning heavy metal resistance in sludge-based ecosystems. They also provide fundamentals for future manipulation of the microbiome to handle metallic toxicants. Moreover, genome-centric metaproteomics was proven to be useful for understanding the adaptation of microbial assemblages under adverse environmental stimuli.

## Supplementary Information

**Additional file 1.** Supplementary information.

**Additional file 2: Supplementary dataset.** The quality metrics, taxonomy, and abundance of MAGs.

**Additional file 3: Supplementary dataset.** The functional annotation, differential expression profile, and potential producer (represented by MAG) of label-free quantified proteins.

**Additional file 4: Supplementary dataset.** The core functioning microbes and their relationships with differentially expressed functions (protein ontologies).

## Data Availability

Raw sequences and mass spectrometry data can be found in NCBI (under BioProject: PRJNA592128) and ProteomeXchange Consortium (iproX repository Project ID: IPX0001891005), respectively. Metadata, source codes, bioinformatic analysis intermediate, and final results for reproducing the results in this study were deposited at https://github.com/DOieGYuan/DPRS_with_HMs. The comprehensive information of the recovered 403 MAGs is provided as Additional file 2. The detailed information for metaproteomics-identified proteins is available in Additional file 3. Core-functioning microbes and their contributed functions are indicated in Additional file 4.
