# Supplementary material for: Revealing taxon-specific heavy metal-resistance mechanisms in denitrifying phosphorus removal sludge using genome-centric metaproteomics
Source: Microbiome. 2021 Mar 22;9:67. doi: 10.1186/s40168-021-01016-x (PMC7986553; doi:10.1186/s40168-021-01016-x)
Supplement: Supplementary file 2 — Additional file 1. Supplementary information. [file 40168_2021_1016_MOESM2_ESM.docx]

**Supplementary Information**

**for**

**Revealing taxon-specific heavy metal resistance mechanisms in denitrifying phosphorus removal sludge using genome-centric metaproteomics**

**Authors:**

Yuan Lin, Liye Wang, Ke Xu, Kan Li, Hongqiang Ren*

**Affiliation:**

State Key Laboratory of Pollution Control and Resource Reuse, School of the Environment, Nanjing University, N.O.163, Xianlin Avenue, Qixia District, Nanjing, Jiangsu, PR China. Email address: hqren@nju.edu.cn

This file includes:

**Supplementary Texts**

Text S1 Composition of synthetic wastewater

Text S2 Analytical methods

Text S3 Metagenomic data processing

Text S4 Metaproteomic sample preparation protocol

Text S5 UHPLC-MS/MS analysis

Text S6 Peptide and protein identification and quantification

Text S7 Enrichment analysis using GSEA

Text S8 MAG co-occurrence network construction using CoNet

**Supplementary Tables**

Table S1 Operating strategy of reactors

Table S2 Reported concentrations of heavy metals in literatures

Table S3 Proteome Discoverer (v2.2) configuration parameters

Table S4 Information for genes of interest

Table S5 Statistics of the equivalence testing

**Supplementary Figures**

Figure S1 Metagenome-based taxonomic profile of denitrifying phosphorus removal sludge (DPRS) microbiome

Figure S2 Abundance profile of denitrifiers

Figure S3 Abundance profile of nitrite oxidizers

Figure S4 High genomic similarity between *Candidatus Accumulibacter* and *Dechloromonas*

Figure S5 The DPRS metaproteomes profiles under heavy metal stresses

Figure S6 Performance of reactors

Figure S7 Shifts in functional profile associating with phosphorus-accumulating organisms (PAOs) at the high-pressure stages

Figure S8 Phosphorus removal and polyphosphate (polyP) accumulating performance after withdrawing heavy metal pressures

Figure S9 Intracellular accumulating rate of heavy metal

Figure S10 Contribution of core functioning microbes to the functional shifts in heavy metal-treated DPRS

Figure S11 EPS composition

Figure S12 Heavy metal adsorptive capacity of extracellular polymeric substance (EPS)

Figure S13 Co-occurrence network of metagenome-assembled genomes (MAGs).

# Supplementary Texts

## Text S1 Composition of synthetic wastewater

Synthetic wastewater contains 439.4 mg/L sodium acetate (CH_3_COONa), 95.5 mg/L ammonium chloride (NH_4_Cl), monopotassium phosphate (1.54 mg/L KH_2_PO_4_), 2.94 mg/L calcium chloride (CaCl_2_·2H_2_O) and 7.35 mg/L magnesium sulfate (MgSO_4_⸱7H_2_O) and 0.6 ml/L trace element solution to reach a composition of 300 mg/L chemical oxygen demand (COD), 25 mg/L ammonia-N, 2 mg/L nitrate-N, 3.5 mg/L orthophosphate-P. The ratio of the ammonia-N and nitrate-N was chosen based on that in real wastewater [1]. The trace element solution contains 1500 mg/L ferric chloride (FeCl_3_⸱6H_2_O), 180 mg/L potassium iodide (KI), 120 mg/L zinc sulfate (ZnSO_4_⸱7H_2_O), 150 mg/L boric acid (H_3_BO_3_), 120 mg/L manganese chloride (MnCl_2_⸱4H_2_O), 150 mg/L cobalt chloride (CoCl_2_⸱6H_2_O), 30 mg/L copper sulfate (CuSO_4_⸱5H_2_O), 60 mg/L sodium molybdate (Na_2_MoO_4_•2H_2_O). Sodium bicarbonate (NaHCO_3_) was used to maintain the pH at 7.4 ± 0.2. The corresponding HM concentrations were adjusted by 10.16 g/L cadmium chloride (CdCl_2_⸱2.5H_2_O), 20.25 g/L nickel chloride (NiCl_2_⸱6H_2_O) and 45.01 g/L sodium chromate (Na_2_CrO_4_·4H_2_O) stock solution respectively. Fresh stock solutions were prepared weekly.

## Text S2 Analytical methods

The measurements of chemical oxygen demand, total nitrogen, ammonia-N, nitrite-N, nitrate-N, total phosphorus, phosphate-P, and mixed liquor suspended solids were based on standard methods [2]. The concentration of polyP was determined the stress-induced assays [3]. Dissolved oxygen (DO), pH and temperature were measured using a pH/DO meter SevenGo Duo (Mettler-Toledo, Zurich, Switzerland). Metal concentrations in total were determined by an inductively coupled plasma optical emission spectrometry iCAP™ 7200 Analyzer (Thermo Fisher Scientific, Waltham, Massachusetts, USA). Hexavalent chromium concentration was analyzed using the 1,5-diphenylcarbazide method accordingly [4]. Since the valence states of Cr changed during operation, we only used the concentration measured by inductively coupled plasma optical emission spectrometry (i.e., total Cr) for convenience. But note that Cr existed in water phase was mostly hexavalent while that accumulated intracellularly was mostly trivalent form. Specific oxygen uptake rate (SOUR) was measured based on the previous method [5], and microbial viability was determined by LIVE/DEAD™ BacLight™ Bacterial Viability Kit (Thermo Fisher Scientific). Sequential extracellular polymeric substance (EPS) extraction to obtain soluble-EPS, loosely bound EPS (LB-EPS) and tightly bound EPS (TB-EPS) was based on the established method [6]. Sludge pellets after EPS extraction were digested to determine the metal amount in sludge according to the standard method proposed by the United States Environmental Protection Agency [7]. The concentrations of polysaccharide (PS), protein (PN) and humic acid (HA) in EPS were determined by the phenol-sulphuric acid method and modified Lowry method, respectively [8].

## Text S3 Metagenomic data processing

**Quality control**

Quality control of raw reads was performed using Trimmomatic (v0.39). Adapters were removed from the paired read using reference "TruSeq3-PE.fa" with seed mismatches<2, palindrome clip threshold>40, simple clip threshold>15, and minAdapterLength>8bp. A sliding window trimming was performed with window size=4, required quality > 15, and minimal length > 50 bp.

**Assembly**

The kmer abundance histogram was estimated, and the set of best k-mer lengths for *de novo* assembly was chosen using KmerGenie (http://kmergenie.bx.psu.edu/). Qualified reads from all samples were co-assembled by MEGAHIT (v1.2.9) with optimized parameters "--k-list 27,33,41,51,61,71,81,91,101,111,121,141 --min-contig-len 1000". The quality of the assembly (e.g., average length and N50) was evaluated by QUAST (v5.0.2) with default parameters. It showed that the resulting 1 048 579 contigs had an average length of 3 600 bp and N50 of 6 130 bp.

**Binning**

Binning was performed using MaxBin2 (v2.2.6) with parameters "-min_contig_length 1000 -max_iteration 55", Metabat2 (v2.13) with parameters "-m 1500", and CONCOCT (v1.1.0) with default parameters. MetaWRAP (v1.3) was used to dereplicate and aggregate the similar bins from these bin sets with parameters "-c 50 -x 10". Potential contaminative contigs with aberrant read depth, GC content, tetranucleotide frequencies or conflicting phylum-level taxonomy were identified using RefineM's (v0.1.2) commands *scaffold_stats*, *outliers*, and *taxon_profile*. Outliers (contigs had GC or tetranucleotide distance outside the 98^th^ percentile of the expected distributions or conflicting phylum-level taxonomy with specific MAGs) were then removed from the corresponding MAGs using command *filter_bins*. Then, the *Reassemble bins* module of MetaWRAP was performed with parameters "-c 50 -x 10 -l 500" to further improve the bins completeness and reduce their contamination. The quality of retrieved MAGs was estimated by checkM (v1.0.13) using *lineage_wf* pipeline. Putative tRNA genes and rRNA genes in each MAG were retrieved using tRNAscan-SE [9] (v2.0.6) and Infernal (v1.1.3) [10] against Rfam database (v14.3) with recommended cutoffs [11]. Only MAGs met with the high-quality (completeness > 90%, contamination < 5%, presence of the 23S, 16S, 5S rRNA genes, and at least 18 tRNAs) or medium-quality (completeness ≥ 50% and contamination < 10%) criteria of the MIMAG standard and had a quality score ≥ 45 (defined as completeness− 5× contamination) were included in the downstream analysis.

**Taxonomic classification**

MAGs were taxonomically classified using GTDB-Tk (v1.02). The corresponding NCBI taxonomy was retrieved based on the genome metadata file provided by GTDB (https://data.ace.uq.edu.au/public/gtdb/data/releases/release95/95.0/auxillary_files/gtdb_vs_ncbi_r95_bacteria.xlsx) and manually verified. A phylogenetic tree of MAGs was constructed based on 92 concatenated core genes alignments following the UBCG pipeline [12] (v3.0). The tree was visualized using iTOL [13] (v5.0) and decorated using the template files downloaded from the official website (https://itol.embl.de/).

**Code availability**

All source codes and step-by-step guidance to reuse these codes are available at https://github.com/DOieGYuan/DPRS_with_HMs.

## Text S4 Metaproteomic sample preparation protocol

**Vessel passivitation**

Tubes for protein extraction are incubated overnight in 5% v/v TWEEN^®^ 20 (T20, Sigma-Aldrich) MS-grade water solution following rinse and immersion in MS-grade water. The rinse and immersion procedures are repeated for twice. This pretreatment can effectively reduce protein loss due to adsorption on the plastic surfaces because T20 can passivate plastic surfaces to reduce protein binding [14].

**Extraction**

(1) Add 10% (w/w) polyvinylpolypyrrolidone (PVPP) to 1 g (wet weight) sludge sample, then grind in liquid nitrogen. PVPP serves as an effective absorbent for humic acids, especially phenolic compounds [15].

(2) Transport ground sludge into a passivated tube, add 1.5 mL Tris-sodium dodecyl sulfate (SDS) buffer (100 mM Tris, 2% SDS, pH=7.5) and 1.5 mL phenol (pH=8.0), followed by vortexing for 1 min. The pH of extraction buffer and phenol can be 6.8-8.5, as previously reported [15-17]. However, more protein yield can be achieved with extraction buffer and phenol at pH 7.5 and 8.0, respectively, according to our experience (data not shown).

(3) Sonicate the suspension with an ultrasonic homogenizer (Biosafer150-96, Biosafer, China) on the ice at a 3 s sonicate with 3 s interval cycle and 45% energy for 2 min to break aggregates and lyse the cells. After shaking at 200 rpm for 1 h, repeat the vortexing and sonication before centrifugation at 14000 g 20 min at 4 ℃.

(4) Upper water phase and lower phenol phase are separately distributed into two clean passivated tubes.

**Purification**

(1) Add an equal volume of MS-grade water to phenol phase and gently vortex for 5 min and then centrifuge at 14000 g for 15 min at 4 ℃. Transport lower purified phenol phase into a new passivated tube.

(2) Add a 5-fold amount of 0.1 M ammonium acetate MS-grade methanol solution to both water phase (to reduce the loss of hydrophilic proteins in water phase [16]) and phenol phase and incubate overnight at -20 ℃ to precipitate protein.

(3) Centrifuge at 14000 g for 20 min at 4 ℃ to pellet precipitated protein and wash (use the force of pipette to resuspend the pellet gently) with pre-chilled MS-grade methanol, 80% MS-grade acetone and 100% MS-grade acetone in sequence. Resuspended protein from water and phenol phase are combined and pooled during the first wash step. Centrifugate at 14000 g 15 min at 4 ℃after each wash step and discard the supernatant.

(4) Air-dry the purified protein and add 1.0 mL 0.2 M triethylammonium bicarbonate (TEAB) buffer containing 10 mM dithiothreitol (DTT), 6 M urea and 1M thiourea. Vortex and gentle shake overnight at 4 ℃ to resuspend the protein. Collect the supernatant after centrifugation at 18000 g for 5 min, and use Compat-Able^™^ BCA Protein Assay Kit (Thermo Fisher Scientific, USA) to determine the protein concentration.

**Digestion**

(1) Add 1 M DTT solution into the protein solution to reach a DTT concentration of 10 mM and incubate for 60 min at 50 ℃ for reducing disulfide crosslinks of cystine to cysteine residues.

(2) For alkylation, add 1 M iodoacetamide to a final concentration of 40 mM, and then incubate for 30 min at 37 ℃ in darkness.

(3) Add sequencing-grade trypsin (Promega, USA) to a final concentration of 2 μg/mL and incubate at 37 ℃. After 12 h, add aliquot (10 μL) MS-grade formic acid to quench the digestion.

**Desalination**

Solid-phase extraction is performed to remove salts and residual detergents which are detrimental to the LC-MS/MS process.

(1) The digested protein sample is diluted by 0.1% MS-grade formic acid before SPE.

(2) 1cc OASIS^®^ hydrophilic-lipophilic balanced cartridge (Waters, USA) is cleaned and conditioned with five 1 mL volumes of MS-grade methanol followed by five 1 mL volumes of 0.1% MS-grade formic acid.

(3) Diluted protein sample passes through the cartridge by gravity flow.

(4) Rinse the cartridge with three 1 mL 0.1% MS-grade formic acid and dry for 10 min under gentle vacuum (approximately 13 mmHg).

(5) The desalinated protein sample is eluted and collected with 5 mL 80% MS-grade acetonitrile.

Finally, dry the sample using the cryogenic centrifugal vacuum evaporator (Eppendorf, Germany), and redissolve the protein sample in 200 μL of 20 mM MS-grade ammonium formate (pH=10) prior to LC-MS/MS analysis.

## Text S5 UHPLC-MS/MS analysis

An analytical column and a Nano-Trap column (pre-column) was filled with ReproSilPur C18-AQ 1.9 μm particles (Dr. Maisch, Germany) and ReproSil-Pur C18-AQ 3 µm particles (Dr. Maisch, Germany), respectively. A sample volume containing 2 µg of total peptides was injected onto the C18 Nano-Trap column (2 cm×100 µm, 3 µm). Peptides were separated on the analytical column (15 cm×150 µm, 1.9 µm), using a 60 min linear gradient from 5 to 100% eluent B (0.1% FA in 80% ACN) in eluent A (0.1% FA in H_2_O) at a flow rate of 600 nL/min. The detailed solvent gradient listed as follows: 5-10% B, 2 min; 10-30% B, 49 min; 30-50% B, 2 min; 50-90% B, 2 min; 90-100% B, 5 min.

Q-Exactive HF-X mass spectrometer was operated in positive polarity mode with a spray voltage of 2.3 kV and a capillary temperature of 320°C. Full MS scans range from 350 to 1500 m/z were acquired at a resolution of 60000 (at 200 m/z) with an automatic gain control (AGC) target value of 3×106 and a maximum ion injection time of 20 ms. The 40 most abundant precursor ions from full MS scan were selected for fragmentation using higher energy collisional dissociation (HCD) fragment analysis at a resolution of 15000 (at 200 m/z) with an AGC target value of 1×10^5^, maximum ion injection time of 45 ms, normalized collision energy of 28%, intensity threshold of 2.2×10^4^, and dynamic exclusion parameter of 20 s.

## Text S6 Peptide and protein identification and quantification

The search parameters of Proteome Discoverer (PD) v2.2 were as follows. Mass tolerances were set to 10 ppm and 0.02 Da for precursor and product ions, respectively. Carbamidomethyl was specified as a fixed modification while oxidation of methionine (M) and acetylation of the N-terminus were specified as variable modifications. A maximum of 2 miscleavage sites was allowed. The detailed configurations of the PD software are available in Table S3.

For protein identification, the protein with at least one unique peptide was identified at FDR < 0.01 both at peptide and protein levels. Proteins containing similar peptides and could not be distinguished based on MS/MS analysis were grouped separately as protein groups. Precursor quantification based on intensity (i.e., area under the curve) was used for label-free quantification.

## Text S7 Enrichment analysis using GSEA

We used GSEA [18] (v.4.0.2) to pre-rank the identified proteins based on their expression differences in the heavy metal-treated groups and the control group. A normalized enrichment score was computed for each protein set to indicate the enrichment of the protein group. False discovery rate (FDR) for *p*-value correction was based on 10 000 times gene set permutation test. Gene sets (protein families) larger than 500 entries or smaller than 15 entries were excluded from the analysis. The similarities between gene sets were calculated based on the Jaccard Overlap combined coefficient (weight factor=0.5 for both Jaccard and Overlap coefficients), and ontologies were linked and clustered with shared edges using the cutoff > 0.25.

## Text S8 MAG co-occurrence network construction using CoNet

MAGs with average abundance > 1 CoPM and occurrence at least in two-thirds of the samples) was selected for co-occurrence network construction using the *data preprocessing* function of CoNet [19] (v2). Then, an ensemble inference was constructed using correlation-based methods "Pearson" and "Spearman", similarity-based method "Mutual Information", and distance-based methods "Bray Curtis" and "Kullback-Leibler". Null distributions were calculated based on 1 000 times randomized permutations, with "edgeScores" as routine and "shuffle_rows" as resampling strategy. Renormalization was enabled to mitigate compositionality biases [20]. *P*-values of an edge were merged into single *p*-values using "Brown's method" with 1 000 bootstraps replications. Then the Benjamini and Hochberg FDR adjusted *p*-value was computed for multiple test correction.

# Supplementary Tables

## Table S1 Operating strategy of reactors

| Procedure | Duration (min) | Operation* | Purpose |
| --- | --- | --- | --- |
| Filling | 5 | Pump 10 (2) L synthetic wastewater into reactor(s) | To feed the reactors with influent |
| Anaerobic | 60 | At the beginning, nitrogen gas is introduced for 5 min | To ensure an anaerobic condition |
| Anoxic | 60 | maintain DO = 0.2-0.5 mg/L by the auto DO control system | To ensure an anoxic condition |
| Aerobic | 120 | maintain DO = 2-4 mg/L by the auto DO control system | To ensure an aerobic condition |
| Discharging | 5 | Remove 300 (60) ml of liquor from Reactor(s) | To ensure an SRT of 16 d and collect the sample(s) for MLSS, SOUR and EPS analysis |
| Settling | 100 | Halt the rotation and aeration | Settle the sludge |
| Decanting | 5 | Withdraw 10 (2) L supernatant from reactor(s) | Finish the present cycle and collect the effluent, resulting a hydraulic retention time of twelve hours |
| Idle | 5 |  | Ready for the next cycle |

*Numbers in parenthesis indicate the operating parameter for daughter reactors

A rotator at 150 rpm was used to homogenize liquor during the reaction period. Throughout the operation, the temperature was maintained at 25 ± 1 ℃ and pH value was 7.4 ± 0.2. Mixed liquor suspended solid (MLSS) was stable at 3600 ± 300 mg with a solid retention time (SRT) of 16 days.

## Table S2 Reported concentrations of heavy metals in literatures

| Metal | Source | Country/Region | Concentration (mg/L) | Reference |
| --- | --- | --- | --- | --- |
| Cd (II) | Pond | China | 1.59-1.66 | [21] |
|  | Stream | China | 0.006 | [21] |
|  | Sewage effluent | Nigeria | 0-0.912 | [22] |
|  | Municipal wastewater | China | 0.54-1.39 | [23] |
|  | Industrial wastewater | India | 1-10 | [24] |
|  | Industrial wastewater | China | 278.8-557.6 | [25] |
|  | Industrial wastewater | Pakistan | 1.48 ± 0.12 | [26] |
|  | Industrial wastewater | India | 4.7 ± 0.1 | [27] |
| Ni (II) | River | India | 0.045-0.24 | [28] |
|  | River | China | 0.009-0.059 | [29] |
|  | Stream | China | 0.54-0.68 | [21] |
|  | Pond | China | 7.31-9.04 | [21] |
|  | Groundwater | Spain | 0.004-0.026 | [30] |
|  | Sewage effluent | Malaysia | 0.193-0.245 | [22] |
|  | Municipal wastewater | NM | 0.1-0.5 | [31] |
|  | Municipal wastewater | China | 0.65-2.41 | [32] |
|  | Industrial wastewater | China | 0.13 | [33] |
|  | Industrial wastewater | China | 0.7 | [34] |
|  | Industrial wastewater | China | 0.8 | [35] |
|  | Industrial wastewater | NM | ≥10 | [31] |
|  | Industrial wastewater | NM | Up to 2950 | [36] |
| Cr (VI) | Municipal wastewater | China | 0.02-1.36 | [32] |
|  | Industrial wastewater | NM | 1-30 | [37] |
|  | Industrial wastewater | China | 18.5 | [34] |
|  | Industrial wastewater | China | 21.03 | [33] |
|  | Industrial wastewater | India | 187.56 | [38] |
|  | Industrial wastewater | Korea | 550 | [4] |

NM, not mentioned.

## Table S3 Proteome Discoverer (v2.2) configuration parameters

| **Processing workflow** | | | |
| --- | --- | --- | --- |
| **Spectrum Selector** | | **Sequest HT** | |
| Precursor Selection | Use MS^1^ Precursor | Protein Database | Customized |
| Use New Precursor Reevaluation | True | Enzyme Name | Trypsin (Full) |
| Use Isotope Pattern in Precursor Reevaluation | True | Max. Missed Cleavage Sites | 2 |
| Lower RT Limit | 0 | Min. Peptide Length | 6 |
| Upper RT Limit | 0 | Max. Peptide Length: | 144 |
| First Scan | 0 | Max. Number of Peptides Reported | 10 |
| Last Scan | 0 | Precursor Mass Tolerance | 10 ppm |
| Lowest Charge State | 0 | Fragment Mass Tolerance | 0.02 Da |
| Highest Charge State | 0 | Use Average Precursor Mass | False |
| Min. Precursor Mass | 350 Da | Use Average Fragment Mass | False |
| Max. Precursor Mass | 5000 Da | Use Neutral Loss a Ions | True |
| Total Intensity Threshold | 0 | Use Neutral Loss b Ions | True |
| Minimum Peak Count | 1 | Use Neutral Loss y Ions | True |
| MS Order | Is Not MS1 | Use Flanking Ions | True |
| Min. Collision Energy | 0 | Weight of a Ions | 0 |
| Max. Collision Energy | 1000 | Weight of b Ions | 1 |
| Scan Type | Is Full | Weight of c Ions | 0 |
| S/N Threshold (FT-only) | 1.5 | Weight of x Ions | 0 |
| Unrecognized Charge Replacements: | Automatic | Weight of y Ions | 1 |
| Unrecognized Mass Analyzer Replacements | ITMS | Weight of z Ions | 0 |
| Unrecognized MS Order Replacements | MS2 | Max. Equal Modifications Per Peptide | 3 |
| Unrecognized Activation Type Replacements | CID | Max. Dynamic Modifications Per Peptide | 4 |
| Unrecognized Polarity Replacements | + | Dynamic Modification | Oxidation (M) |
| Unrecognized MS Resolution@200 Replacements | 60000 | Dynamic Modifications (protein terminus) | Acetyl (N-Terminus) |
| Unrecognized MS^n^ Resolution@200 Replacements | 30000 | Static Modification | Carbamidomethyl (C) |
| Precursor Clipping Range Before | 2.5 Da | **Minora Feature Detector** | |
| Precursor Clipping Range After | 5.5 Da | Min. Trace Length | 5 |
| **Percolator** | | Min. # Isotopes | 2 Peaks |
| Maximum Delta C^n^ | 0.05 | Max. ΔRT of Isotope Pattern Multiplets [min] | 0.2 |
| Maximum Rank | 0 | PSM Confidence At Least | High |
| Target FDR (Strict) | 0.01 |  |  |
| Target FDR (Relaxed) | 0.05 |  |  |
| Validation based on | q-Value |  |  |
| **Consensus workflow** | | | |
| **MSF Files** | | **PSM Grouper** | |
| Spectra to Store | Identified or Quantified | Site Probability Threshold | 75 |
| Feature Traces to Store | All | **Peptide Validator** | |
| Merge Mode | Globally by Search Engine Type | Validation Mode | Automatic (Control peptide level error rate if possible) |
| File Limit for Automatic Merge | 10 | Target FDR (Strict) for PSMs | 0.01 |
| Reported FASTA Title Lines | Best match | Target FDR (Relaxed) for PSMs | 0.05 |
| Title Line Rule | standard | Target FDR (Strict) for Peptides | 0.01 |
| Maximum Delta Cn | 0.05 | Target FDR (Relaxed) for Peptides | 0.05 |
| Maximum Rank | 0 | Validation Based on | q-Value |
| Maximum Delta Mass | 0 ppm | Use Concatenated FDR Calculation for PSM Level FDR Calculation Based on Score | False |
|  |  | Reset Confidences for Nodes without Decoy Search (Fixed score thresholds) | False |
| **Peptide and Protein Filter** | | **Protein Grouping** | |
| Peptide Confidence At Least | High | Apply strict parsimony principle | True |
| Keep Lower Confident PSMs | False | **Peptide in Protein Annotation** | |
| Minimum Peptide Length | 6 | Annotate Flanking Residues of the Peptide | True |
| Remove Peptides Without Protein Reference | False | Number Flanking Residues in Connection Tables | 1 |
| Minimum Number of Peptide Sequences | 2 | Protein Modifications Reported | Only for Master Proteins |
| Count Only Rank 1 Peptides | False | Modification Sites Reported | All And Specific |
| Count Peptides Only for Top Scored Protein | False | Minimum PSM Confidence | High |
| **Protein Scorer** | | Report Only PTMs | False |
| No parameters |  | Protein Positions for Peptides | Only for Master Proteins |
| **Protein FDR Validator** | | **Precursor Ions Quantifier** | |
| Target FDR (Strict) | 0.01 | Peptides to Use | Unique |
| Target FDR (Relaxed) | 0.05 | Consider Protein Groups for Peptide Uniqueness | True |
| **Protein Marker** | | Reject Quan Results with Missing Channels | False |
| As Species Map | False | Precursor Abundance Based On | Intensity |
| As Species Names | False | Min. Replicate Features | 0 |
| **Feature Mapper** | | Normalization Mode | Total Peptide Amount |
| Perform RT Alignment | True | Scaling Mode | On All Average |
| Maximum RT Shift [min] | 10 | Ratio Calculation | Pairwise Ratio Based |
| Mass Tolerance | 10 ppm | Maximum Allowed Fold Change | 100 |
| Parameter Tuning | Coarse | Imputation Mode | None |
| RT Tolerance [min] | 0 | Hypothesis Test | ANOVA (Background Based) |
| Mass Tolerance | 0 ppm | 1^st^ Fold Change Threshold | 2 |
| Min. S/N Threshold | 5 | 2^nd^ Fold Change Threshold | 4 |
|  |  | 3^rd^ Fold Change Threshold | 6 |
|  |  | 4^th^ Fold Change Threshold | 8 |
|  |  | 5^th^ Fold Change Threshold | 10 |

## Table S4 Information for genes of interest

| Gene | Source | Function |
| --- | --- | --- |
| *amo*A | Pfam: PF02461 | Ammonia to hydroxylamine. |
| *amo*B | Pfam: PF04744 |  |
| *amo*C | Pfam: PF04896 |  |
| *hao* | IPG: hydroxylamine oxidoreductase | Hydroxylamine oxidation to nitric oxide. |
| *nxr*A | IPG: nitrite oxidoreductase alpha subunit | Nitrite oxidation to nitrate. |
| *nxr*B | IPG: nitrite oxidoreductase beta subunit |  |
| *nar*G | Pfam: PF02665 | Nitrate reduction to nitrite (dissimilatory). |
| *nar*H | Pfam: PF14711 |  |
| *nap*A | IPG: napA |  |
| *nas*A | IPG: assimilatory nitrate reductase alpha | Nitrate reduction to nitrite (assimilatory). |
| *nas*B | IPG: assimilatory nitrate reductase beta |  |
| *nir*K | IPG: nirK | Nitrite reduction to nitric oxide |
| *nir*S | IPG: nirS |  |
| *nrf*A | IPG: nrfA | Nitrite reduction to ammonium. |
| *nrf*H | IPG: nrfH |  |
| *ppk*1 | Pfam: PF02503 | Phosphate to polyphosphate. |
| *ppk*2 | Pfam: PF03976 |  |
| *ppx* | Pfam: PF02541 | Remove phosphate from polyphosphate. |

The database is available at https://figshare.com/articles/DPRS_with_HMs/12318458.

IPG means the Identical Protein Groups engine in NCBI.

## Table S5 Statistics of the equivalence testing

| Stage* | Comparison | Period | n | MeanRCd | MeanRCK | Equivalence test | Null Hypothesis Test |
| --- | --- | --- | --- | --- | --- | --- | --- |
| LP | R_Cd_ vs. R_CK_ | d1-d48 | 26 | 76.52±3.88 | 75.81±2.99 | t(46.95)=-2.866, *p*=0.0031 | t(46.95)=0.739, *p*=0.464 |
| LP | R_Ni_ vs. R_CK_ | d4-d48 | 23 | 74.42±3.59 | 75.75±3.17 | t(43.34)=2.059, *p*=0.0228 | t(43.34)=-1.332, *p*=0.190 |
| LP | R_Cr_ vs. R_CK_ | d6-d48 | 22 | 76.02±3.16 | 75.73±3.24 | t(41.97)=-3.016, *p*=0.00217 | t(41.97)=0.301, *p*=0.765 |
| MP | R_Cd_ vs. R_CK_ | d56-d106 | 26 | 79.08±3.12 | 79.19±3.13 | t(50)=3.479, *p*=0.000527 | t(50)=-0.127, *p*=0.900 |
| MP | R_Ni_ vs. R_CK_ | d50-d106 | 29 | 77.38±2.81 | 78.79±3.21 | t(55.04)=2.028, *p*=0.0237 | t(55.04)=-1.780, *p*=0.0806 |
| MP | R_Cr_ vs. R_CK_ | d98-d152 | 28 | 81.20±4.13 | 81.57±2.41 | t(43.48)=3.332, *p*=0.000883 | t(43.48)=-0.409, *p*=0.684 |

Cohen's d=1.0, α=0.05.

*LP, low-pressure stage; MP, medium-pressure stage.

# Supplementary Figures


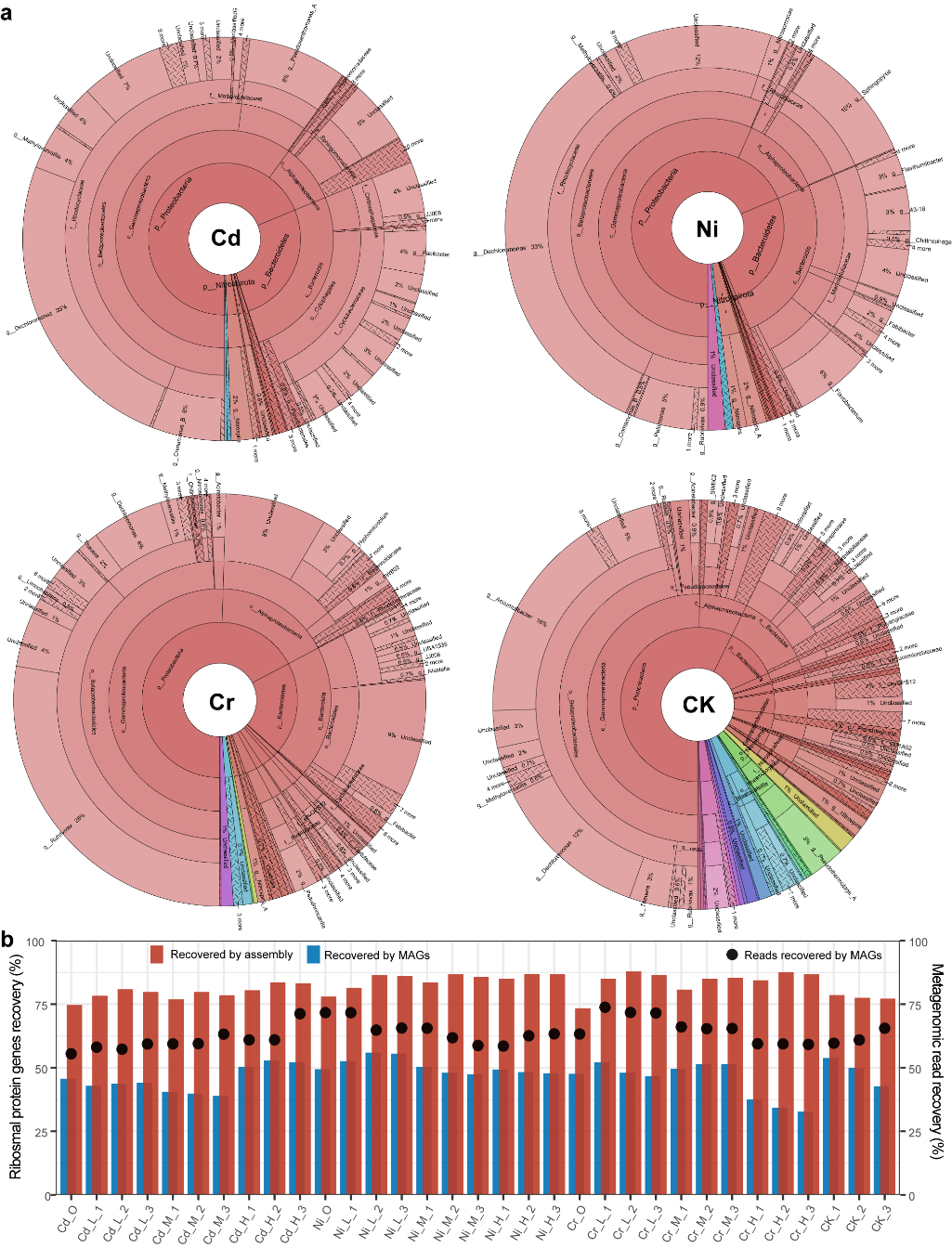


## Figure S1 Metagenome-based taxonomic profile of denitrifying phosphorus removal sludge (DPRS) microbiome. a Taxonomy based on singleM-extracted ribosomal protein genes. Only profiles at the high-pressure stages of heavy metal-treated DPRS and the control DPRS are presented. Full data in the form of interactive vector graph is available at Figshare (figshare.com/articles/DPRS_with_HMs/12318458). b The recoveries (in percentage) of ribosomal protein genes (estimated by singleM) and metagenomic reads (estimated by coverM) by metagenome-assembled genomes (MAGs).


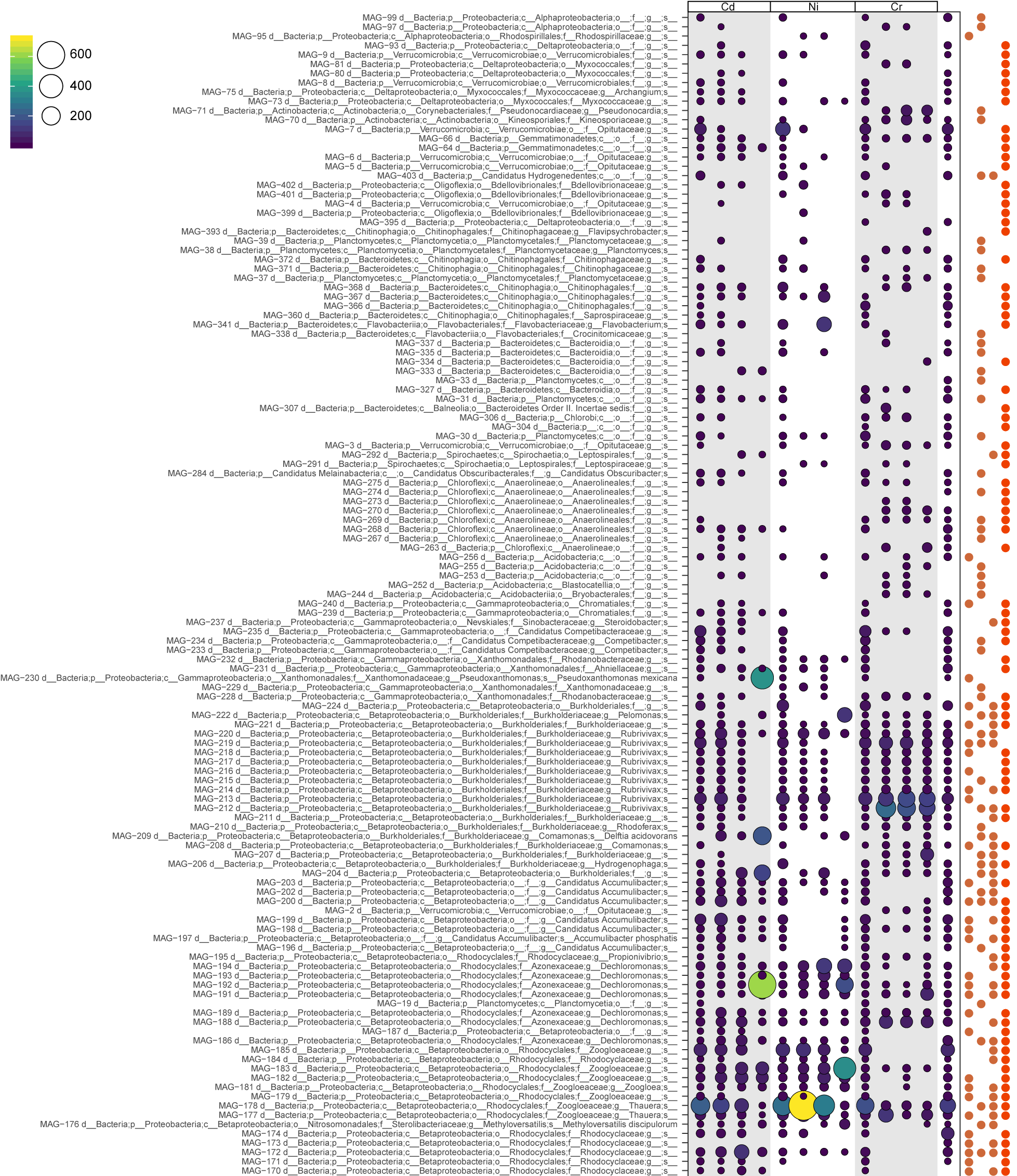


##
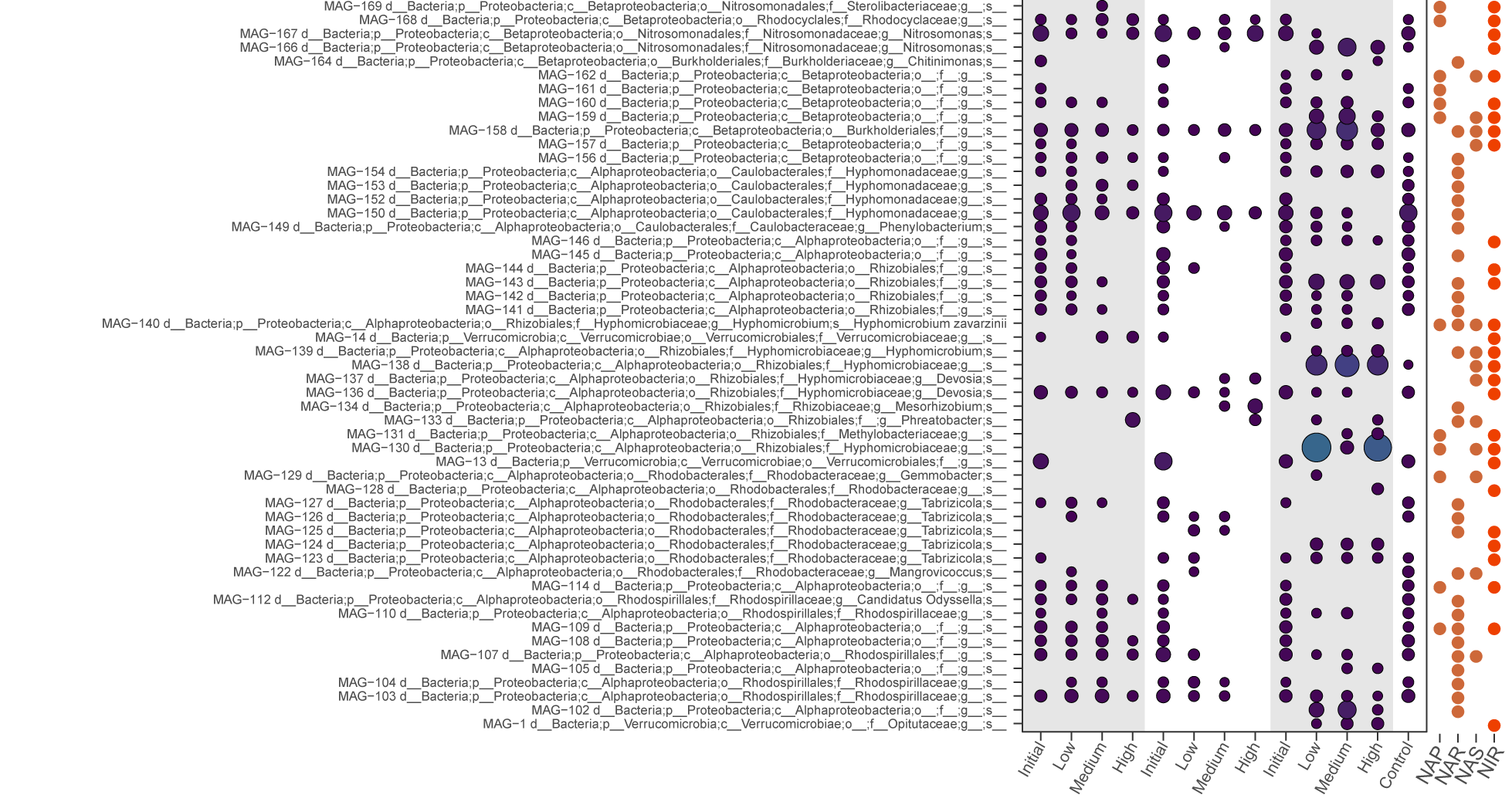
Figure S2 Abundance profile of denitrifiers. Metagenome-assembled genomes (MAGs) with abundance < 1 CoPM were omitted from the graph. Presence of node on the right panel indicate the presence of corresponding functional gene (NAR membrane-bound nitrate reductase, NAP periplasmic dissimilatory nitrate reductases, NAS assimilatory nitrate reductase, NIR nitrite reductase) in the genome.


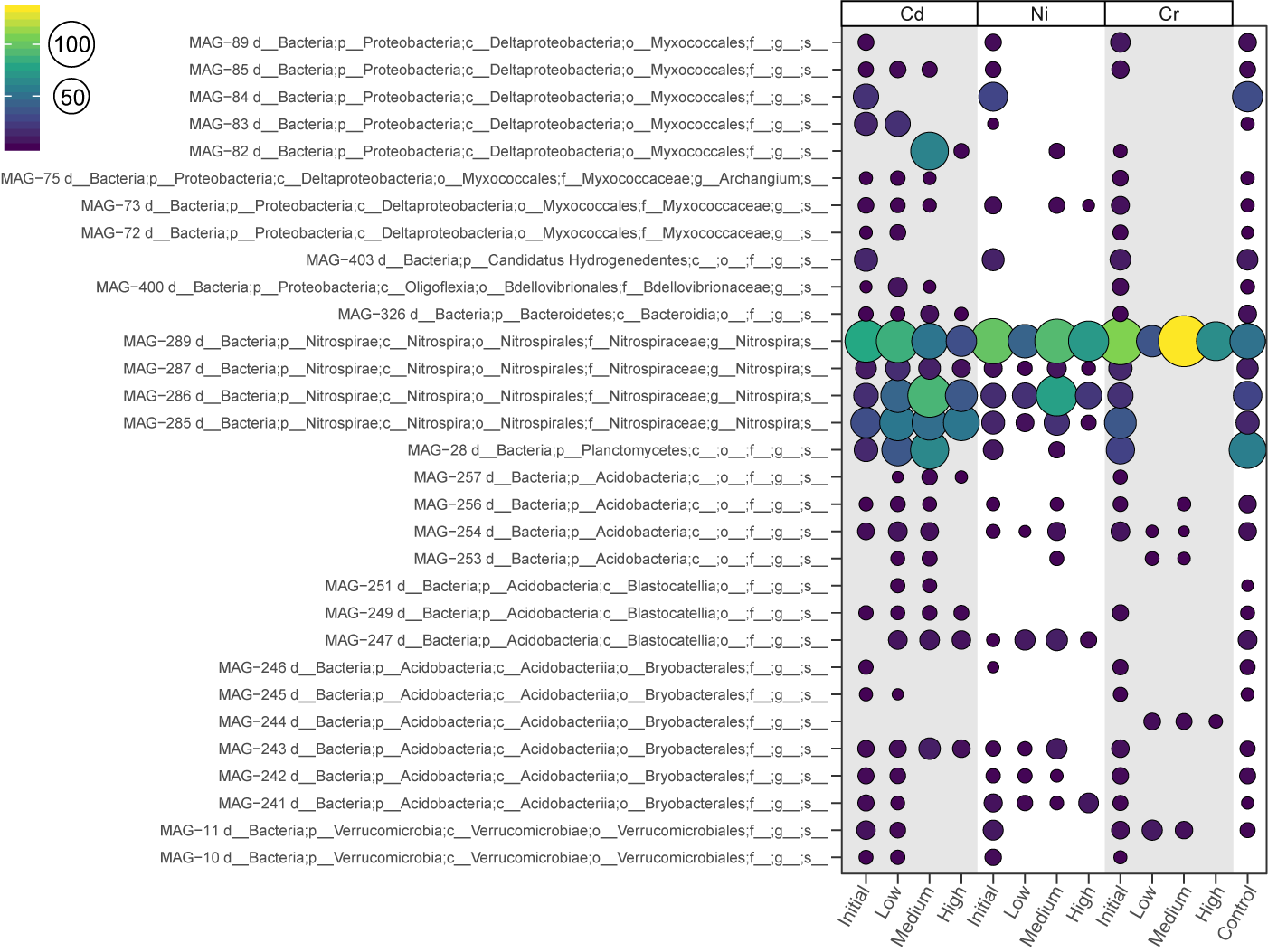


## Figure S3 Abundance profile of nitrite oxidizers. Metagenome-assembled genomes (MAGs) with abundance < 1 CoPM were omitted from the graph.


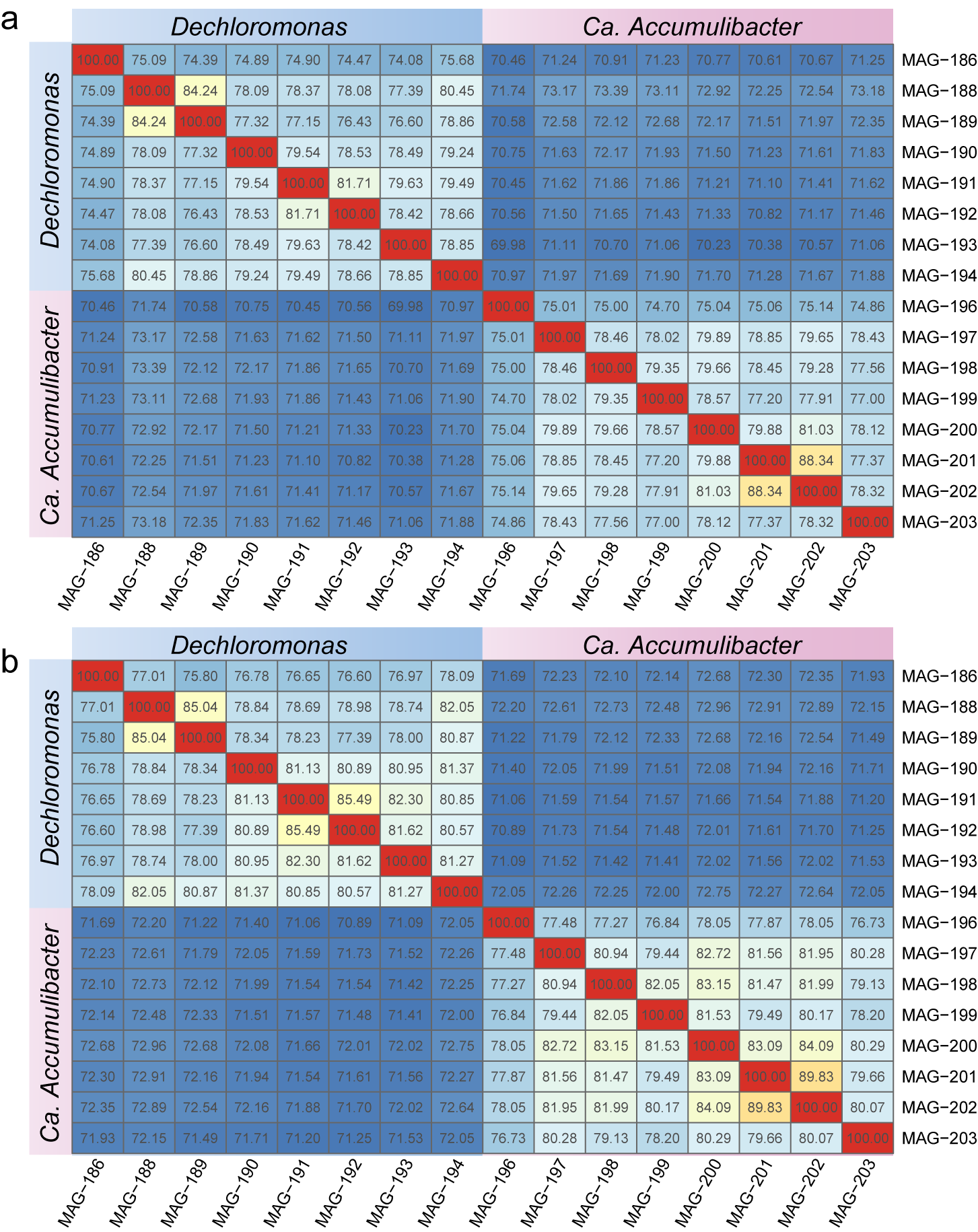


## Figure S4 High genomic similarity between *Candidatus Accumulibacter* and *Dechloromonas*. a Average nucleotide identity (ANI) and b Amino acid identity (AAI).


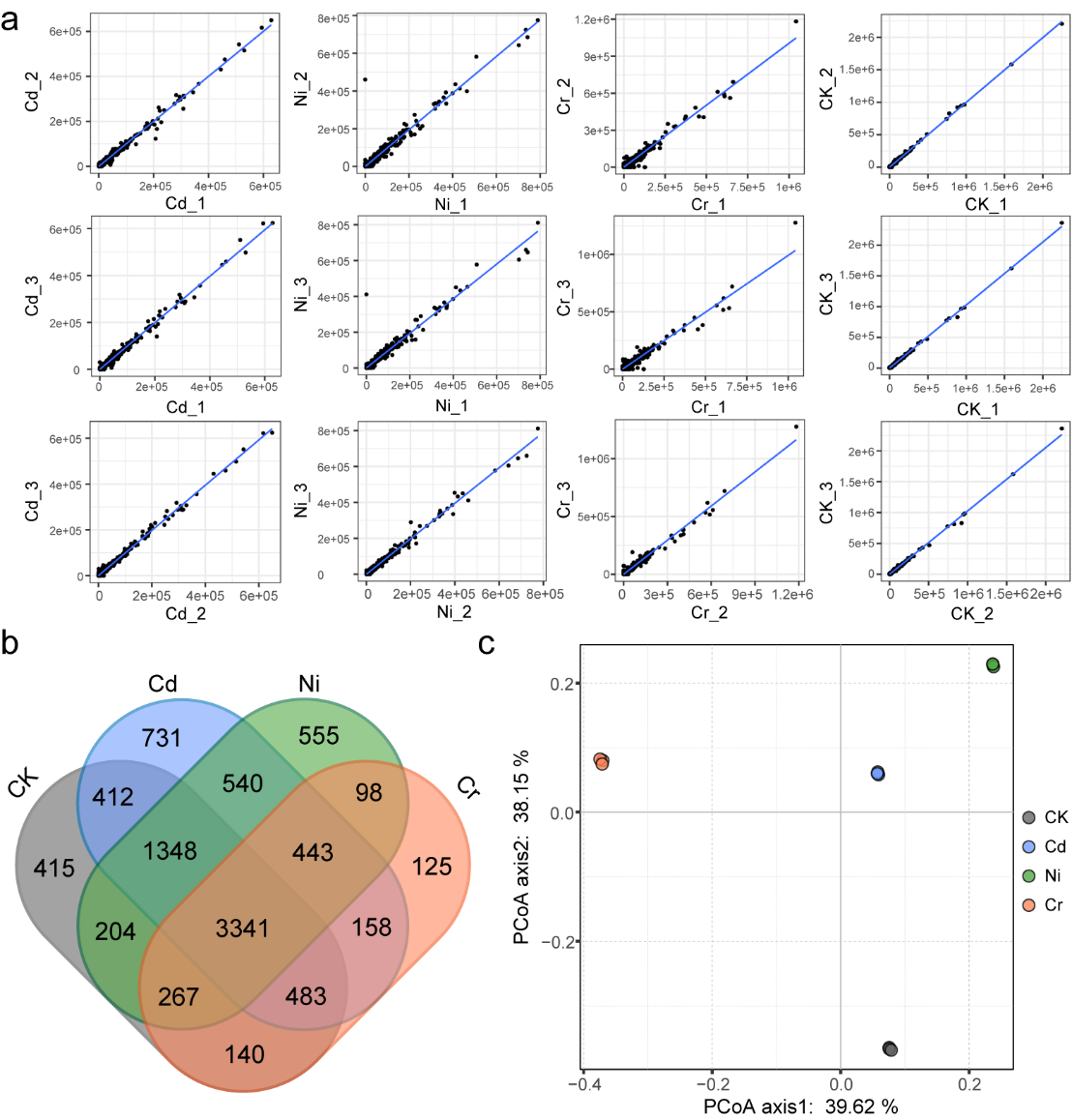


## Figure S5 The DPRS metaproteomes profiles under heavy metal stresses. a The correlation of label-free quantified protein abundances between biological replicates. b Venn graph displays the number of proteins identified in each reactor. c Principal component analysis based on the Bray-Curtis distance of metaproteome compositions.


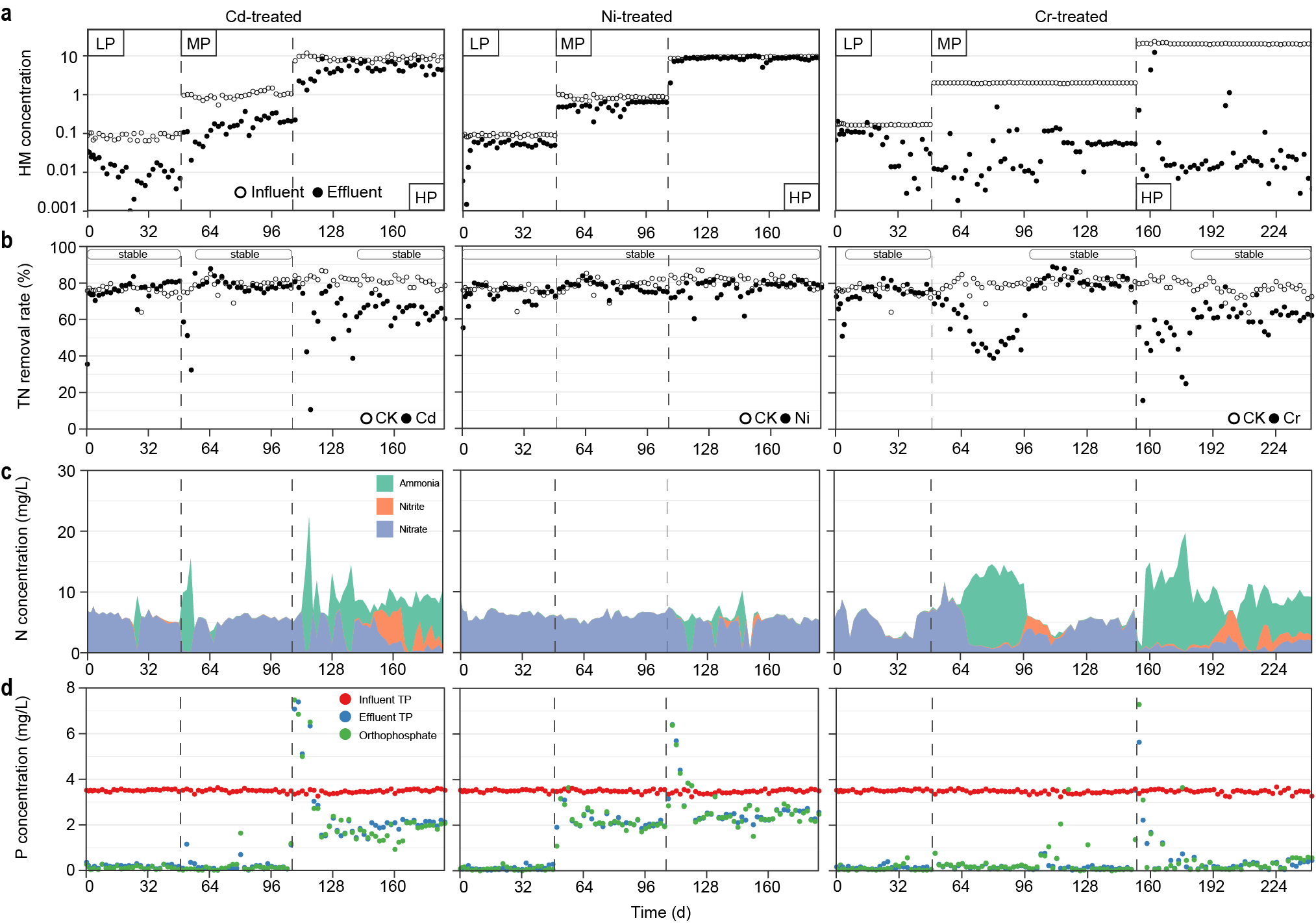


## Figure S6 Performance of reactors. a Heavy metal concentration in the influent/effluent. b Total nitrogen removal rate. c Concentration of different speciation (in different colors) of nitrogen. d Total phosphorus concentration in the influent/effluent and orthophosphate concentration in the effluent.


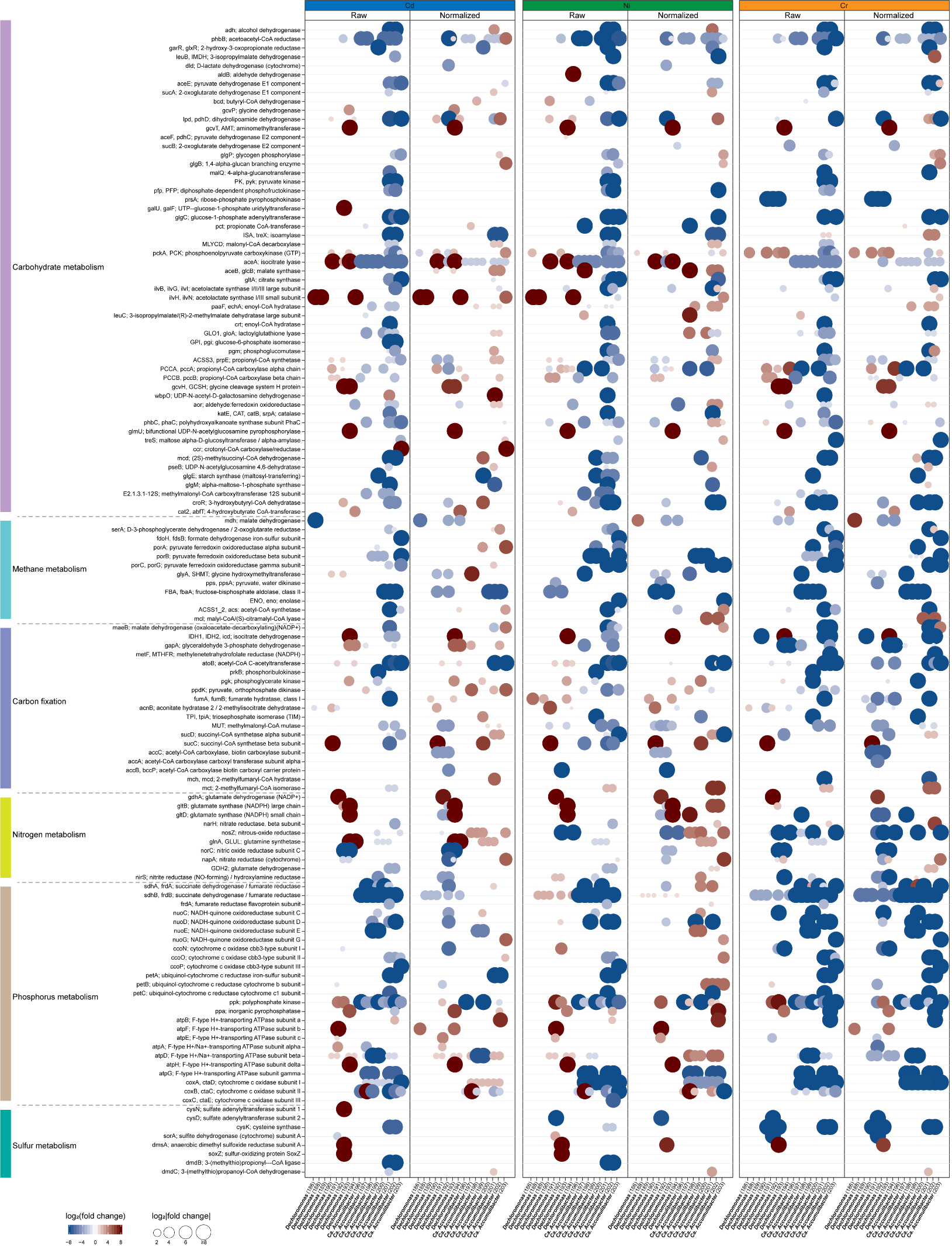


## Figure S7 Shifts in functional profile associating with phosphorus-accumulating organisms (PAOs) at the high-pressure stages. The fold change of protein expression is calculated based on its raw and normalized abundance in the heavy metal-treated group compared with that in the control group. For conciseness, only these under “energy metabolism” category in KEGG BRITE and with adjusted *p*-value < 0.05 and log2 fold change > 1 or < -1 are shown. (Full data are given in additional file 3)


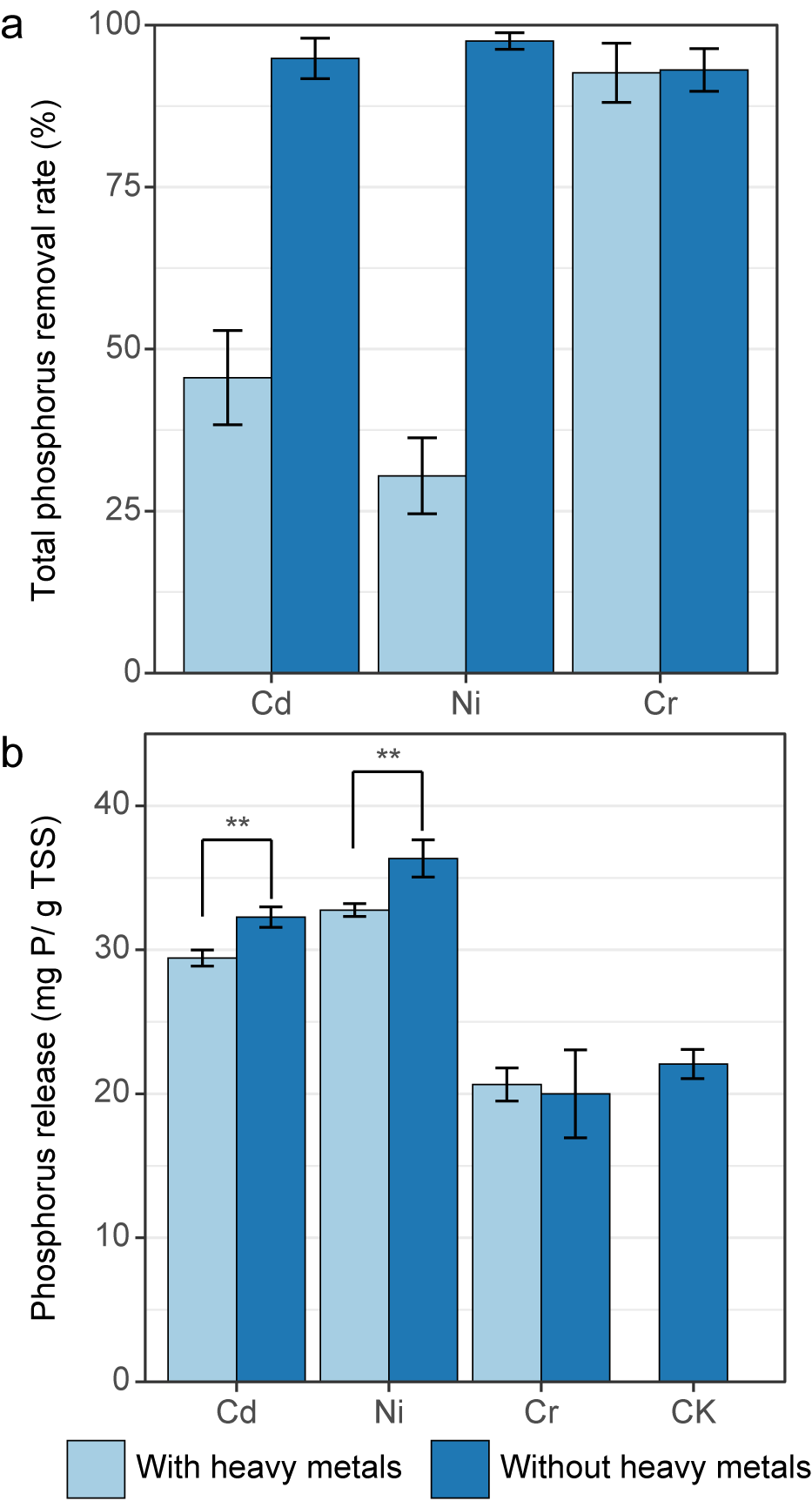


## Figure S8 Phosphorus removal and polyphosphate (polyP) accumulating performance after withdrawing heavy metal pressures. a Phosphorus removal rate in batch experiments. b Amount of accumulated polyP in DPRS after five operating cycles without sludge discharge in batch experiments. Error bars indicate standard errors calculated from five replicates. Significance test is based on the Student’s t-test, **: *p* < 0.01.


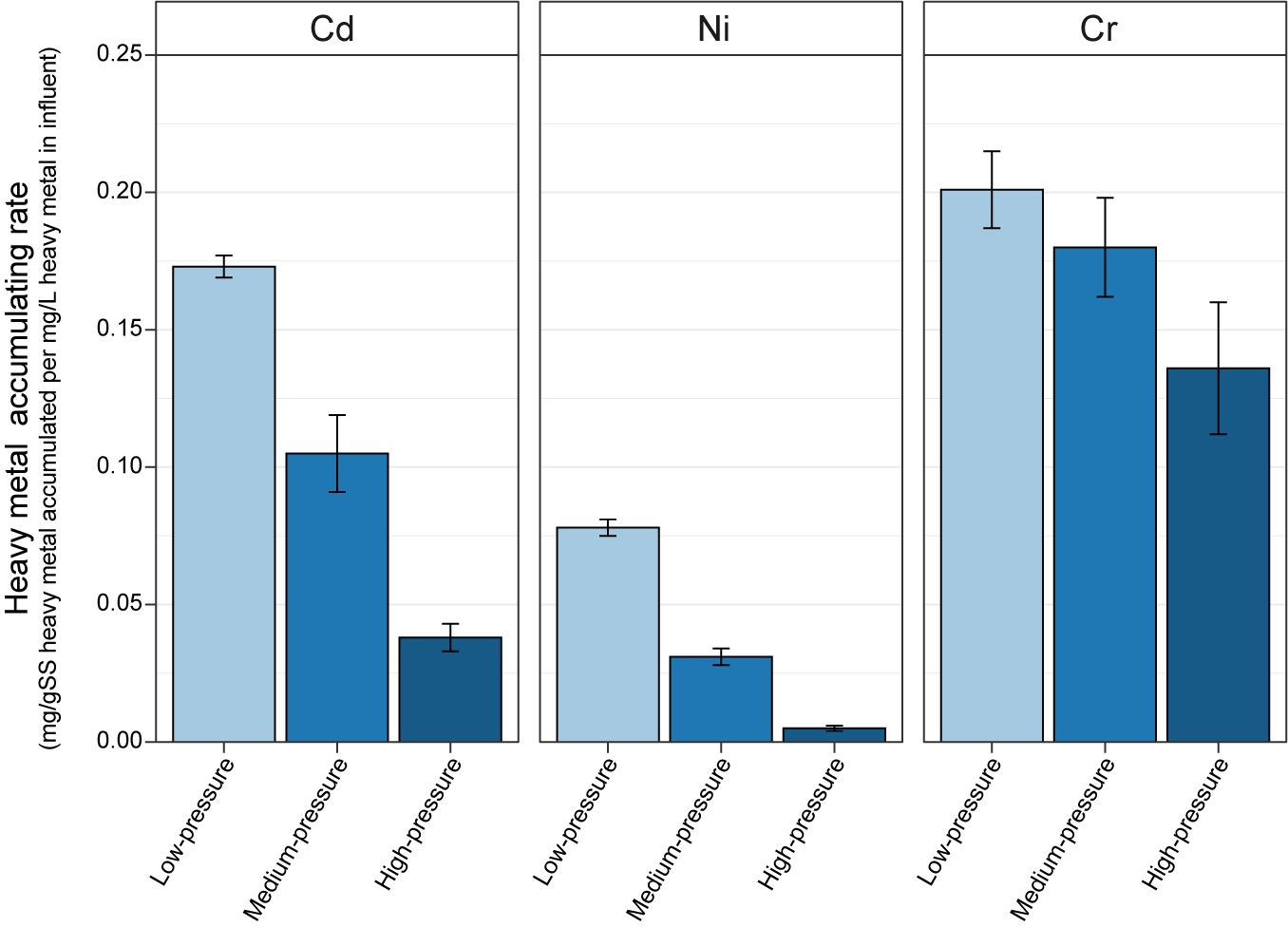


## Figure S9 Intracellular accumulating rate of heavy metal. The accumulating rate is calculated using: $\boldsymbol{Accumulating rate=}\frac{\boldsymbol{C}_{\boldsymbol{t}}\boldsymbol{-}\boldsymbol{C}_{\boldsymbol{o}}}{\boldsymbol{tc}}$. Where *C_t_* is the determined heavy metal concentration in sludge at the end of each stage, *C_o_* is the determined heavy metal concentration in sludge at the initial of each stage, *t* is the operating time (day) between two testing events, and *c* is the heavy metal concentration in the influent. Error bars indicate the standard errors calculated from nine replicates.

##
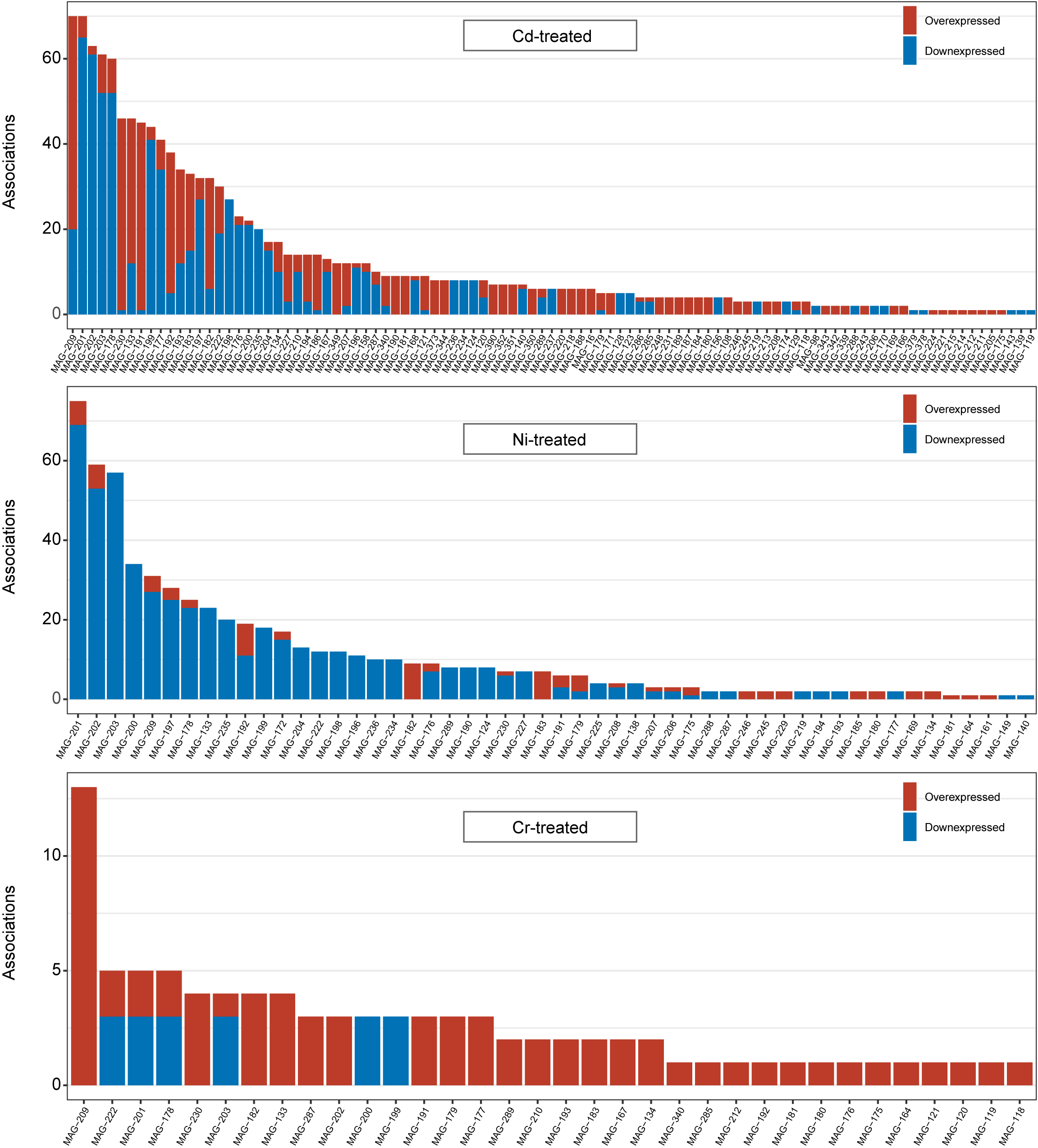
Figure S10 Contribution of core functioning microbes to the functional shifts in heavy metal-treated DPRS. The linkages associating to the up-expressed and down-expressed ontologies are colored in red and blue, respectively.


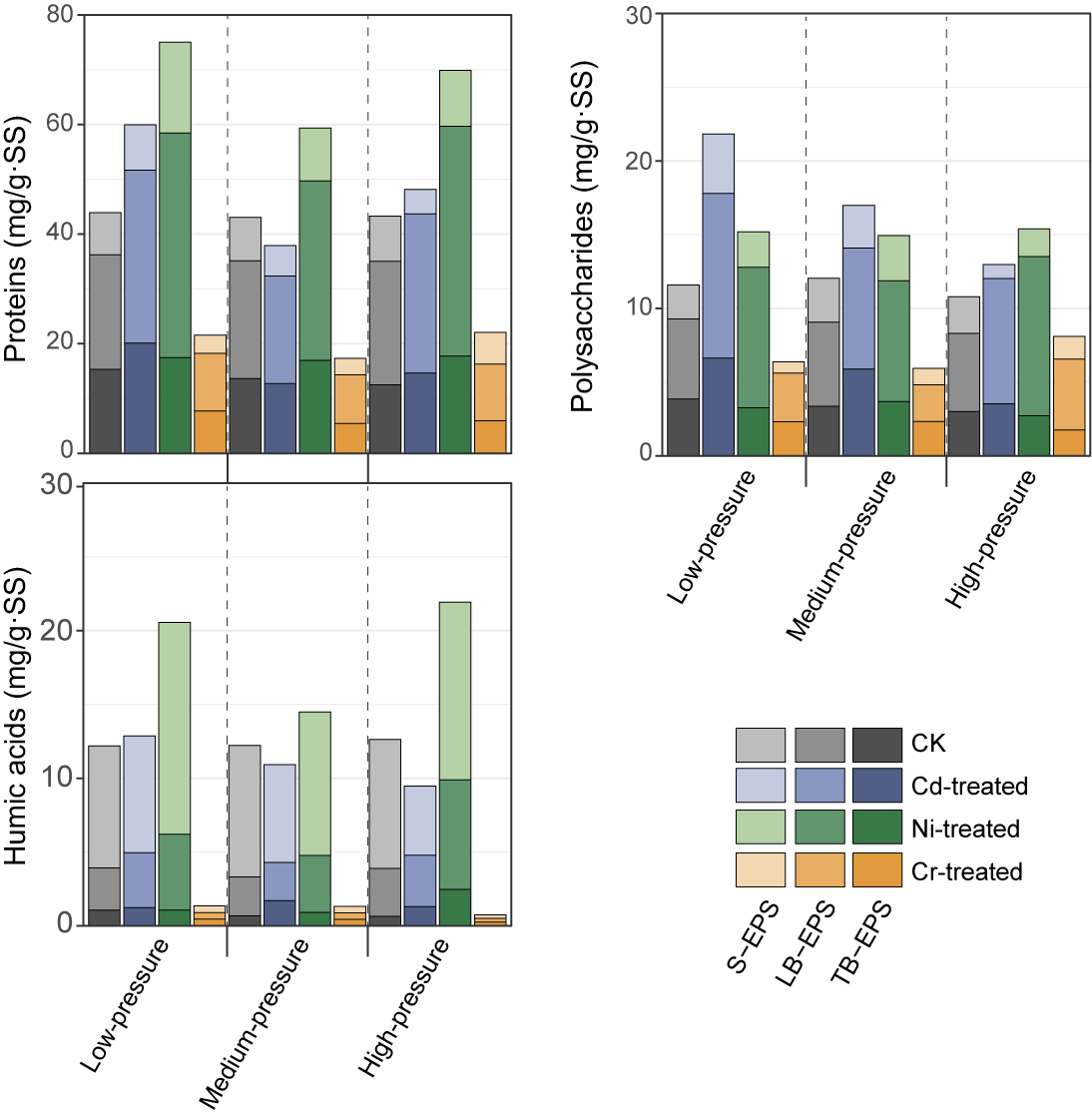


## Figure S11 EPS composition. S-EPS, LB-EPS and TB-EPS refers to soluble-EPS, loosely bound EPS, and tightly bound EPS, respectively.


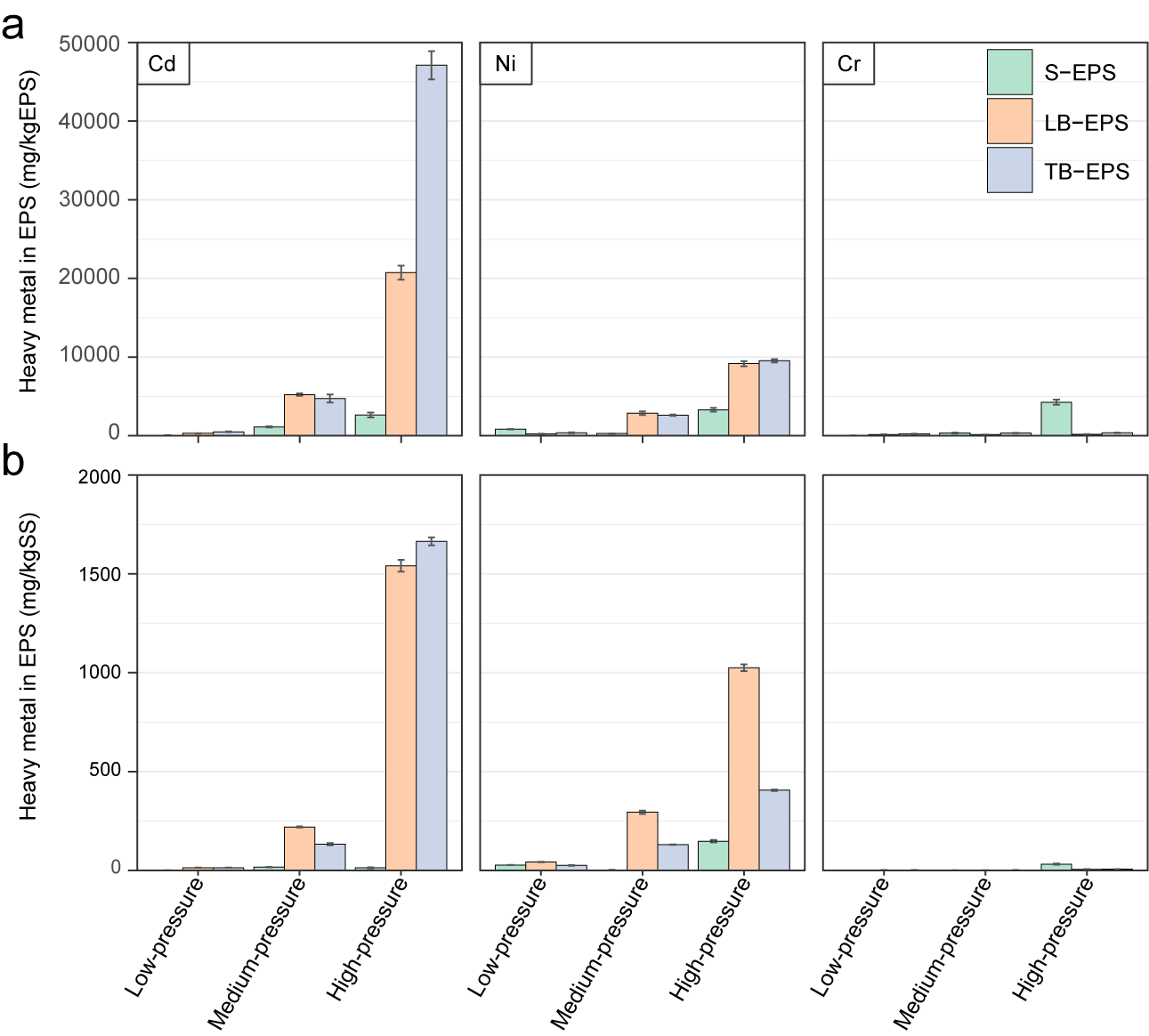


## Figure S12 Heavy metal adsorptive capacity of extracellular polymeric substance (EPS). a Adsorption capacity represented as microgram adsorbed heavy metals per kg EPS. b The total microgram adsorbed heavy metals per kg sludge. Error bars indicate standard deviations from nine replicates. EPS was stratified as soluble EPS (S-EPS), loosely bounded EPS (LB-EPS) and tightly bounded EPS (TB-EPS).


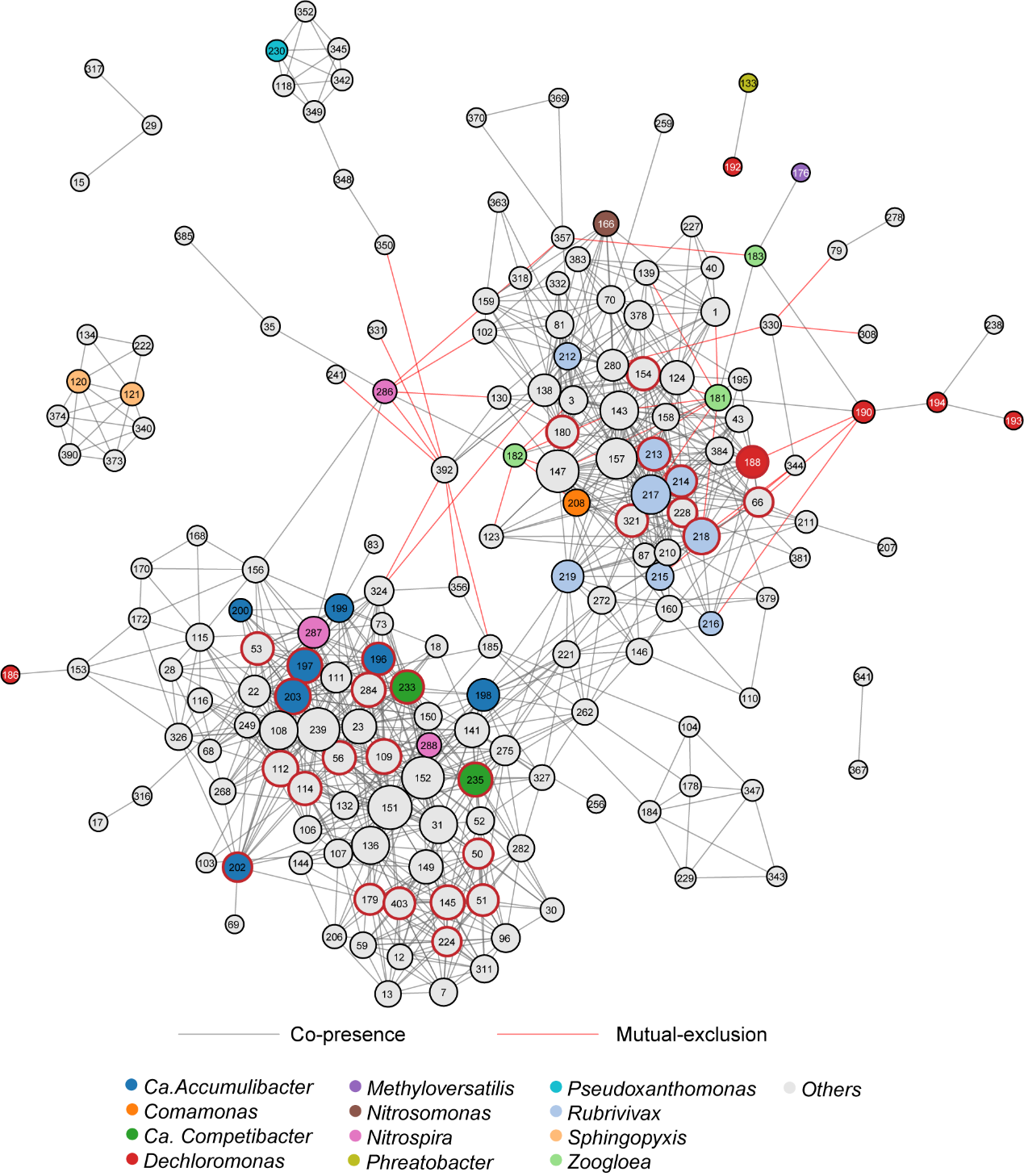


## Figure S13 Co-occurrence network of metagenome-assembled genomes (MAGs). Numbers in circles indicate the IDs of MAGs (e.g., 1 means MAG-1). Edges refer to interspecific interactions estimated by CoNet. Node size is positively related to the degree of corresponding MAG. Nodes with bold red border are keystone species (degree ≥ 15 and betweenness ≤ 250).

# Supplementary References

1. Krustok I, Odlare M, Truu J and Nehrenheim E. Inhibition of nitrification in municipal wastewater-treating photobioreactors: Effect on algal growth and nutrient uptake*.* Bioresour Technol. 2016; 202: 238-243.

2. APHA, *Standard Methods for the Examination of Water and Wastewater*. 20 ed American Public Health Association. Washington DC, USA 2005.

3. Feng C, Welles L, Zhang X, Pronk M, de Graaff D, and van Loosdrecht M. Stress-induced assays for polyphosphate quantification by uncoupling acetic acid uptake and anaerobic phosphorus release*.* Water Res. 2019: 115228.

4. Kim C, Lee C R, Song Y E, Heo J, Choi S M, Lim D-H, et al. Hexavalent chromium as a cathodic electron acceptor in a bipolar membrane microbial fuel cell with the simultaneous treatment of electroplating wastewater*.* Chem Eng J. 2017; 328: 703-707.

5. Surmacz-Gorska J, Gernaey K, Demuynck C, Vanrolleghem P and Verstraete W. Nitrification monitoring in activated sludge by oxygen uptake rate (OUR) measurements*.* Water Res. 1996; 30(5): 1228-1236.

6. Yu G-H, He P-J, Shao L-M and He P-P. Stratification Structure of Sludge Flocs with Implications to Dewaterability*.* Environ Sci Technol. 2008; 42(21): 7944-7949.

7. EPA U S, *Method 3050B: Acid Digestion of Sediments, Sludges, and Soils*. 1996.

8. Huang H, Lin Y, Peng P, Geng J, Xu K, Zhang Y, et al. Calcium ion- and rhamnolipid-mediated deposition of soluble matters on biocarriers*.* Water Res. 2018; 133: 37-46.

9. Lowe T M and Eddy S R. tRNAscan-SE: A Program for Improved Detection of Transfer RNA Genes in Genomic Sequence*.* Nucleic Acids Res. 1997; 25(5): 955-964.

10. Nawrocki E P, Kolbe D L and Eddy S R. Infernal 1.0: inference of RNA alignments*.* Bioinformatics. 2009; 25(10): 1335-1337.

11. Kalvari I, Argasinska J, Quinones-Olvera N, Nawrocki E P, Rivas E, Eddy S R, et al. Rfam 13.0: shifting to a genome-centric resource for non-coding RNA families*.* Nucleic Acids Res. 2017; 46(D1): D335-D342.

12. Na S-I, Kim Y O, Yoon S-H, Ha S-m, Baek I, and Chun J. UBCG: Up-to-date bacterial core gene set and pipeline for phylogenomic tree reconstruction*.* Journal of Microbiology. 2018; 56(4): 280-285.

13. Letunic I and Bork P. Interactive Tree Of Life (iTOL) v4: recent updates and new developments*.* Nucleic Acids Res. 2019; 47(W1): W256-W259.

14. Erde J, Loo R R O and Loo J A. Enhanced FASP (eFASP) to Increase Proteome Coverage and Sample Recovery for Quantitative Proteomic Experiments*.* J Proteome Res. 2014; 13(4): 1885-1895.

15. Isaacson T, Damasceno C M, Saravanan R S, He Y, Catalá C, Saladié M, et al. Sample extraction techniques for enhanced proteomic analysis of plant tissues*.* Nat Protoc. 2006; 1(2): 769.

16. Wöhlbrand L, Feenders C, Nachbaur J, Freund H, Engelen B, Wilkes H, et al. Impact of Extraction Methods on the Detectable Protein Complement of Metaproteomic Analyses of Marine Sediments*.* Proteomics. 2017; 17(22): 1700241-n/a.

17. Leary D H, Hervey W J, Deschamps J R, Kusterbeck A W and Vora G J. Which metaproteome? The impact of protein extraction bias on metaproteomic analyses (Reprinted from Molecular and Cellular Probes, vol 27, pg 193-199, 2014)*.* Mol Cell Probes. 2014; 28(2-3): 51-57.

18. Subramanian A, Tamayo P, Mootha V K, Mukherjee S, Ebert B L, Gillette M A, et al. Gene set enrichment analysis: A knowledge-based approach for interpreting genome-wide expression profiles*.* Proc Natl Acad Sci U S A. 2005; 102(43): 15545-15550.

19. Faust K and Raes J. CoNet app: inference of biological association networks using Cytoscape [version 2; peer review: 2 approved]*.* F1000Research. 2016; 5(1519).

20. Faust K, Sathirapongsasuti J F, Izard J, Segata N, Gevers D, Raes J, et al. Microbial Co-occurrence Relationships in the Human Microbiome*.* PLoS Comput Biol. 2012; 8(7): e1002606.

21. Wu Q, Leung J Y S, Geng X, Chen S, Huang X, Li H, et al. Heavy metal contamination of soil and water in the vicinity of an abandoned e-waste recycling site: Implications for dissemination of heavy metals*.* Sci Total Environ. 2015; 506-507: 217-225.

22. Al-Gheethi A A S, Lalung J, Noman E A, Bala J D and Norli I. Removal of heavy metals and antibiotics from treated sewage effluent by bacteria*.* Clean Technologies and Environmental Policy. 2015; 17(8): 2101-2123.

23. Xu Q X, Li X M, Ding R R, Wang D B, Liu Y W, Wang Q L, et al. Understanding and mitigating the toxicity of cadmium to the anaerobic fermentation of waste activated sludge*.* Water Res. 2017; 124: 269-279.

24. Manasi, Rajesh V, Santhana Krishna Kumar A and Rajesh N. Biosorption of cadmium using a novel bacterium isolated from an electronic industry effluent*.* Chem Eng J. 2014; 235: 176-185.

25. Hu S, Yan L, Chan T and Jing C. Molecular Insights into Ternary Surface Complexation of Arsenite and Cadmium on TiO2*.* Environ Sci Technol. 2015; 49(10): 5973-5979.

26. Khan S, Shamshad I, Waqas M, Nawab J and Ming L. Remediating industrial wastewater containing potentially toxic elements with four freshwater algae*.* Ecological Engineering. 2017; 102: 536-541.

27. Bhateria R and Dhaka R. Optimization and statistical modelling of cadmium biosorption process in aqueous medium by Aspergillus niger using response surface methodology and principal component analysis*.* Ecological Engineering. 2019; 135: 127-138.

28. Aktar M W, Paramasivam M, Ganguly M, Purkait S and Sengupta D. Assessment and occurrence of various heavy metals in surface water of Ganga river around Kolkata: a study for toxicity and ecological impact*.* Environ Monit Assess. 2010; 160(1-4): 207-213.

29. Xu L, Wang T Y, Wang J H and Lu A X. Occurrence, speciation and transportation of heavy metals in 9 coastal rivers from watershed of Laizhou Bay, China*.* Chemosphere. 2017; 173: 61-68.

30. Teijon G, Candela L, Tamoh K, Molina-Diaz A and Fernandez-Alba A R. Occurrence of emerging contaminants, priority substances (2008/105/CE) and heavy metals in treated wastewater and groundwater at Depurbaix facility (Barcelona, Spain)*.* Sci Total Environ. 2010; 408(17): 3584-3595.

31. Yang Q, Sun J, Wang D, Wang S, Chen F, Yao F, et al. Effect of nickel on the flocculability, settleability, and dewaterability of activated sludge*.* Bioresour Technol. 2017; 224: 188-196.

32. Wen Q X, Wang Q, Chen Z Q, Li X Q and Tian Y. Effects of Cu2+ on biological process of wastewater treatment plant (WWTP) in electroplating industrial park*.* Desalination and Water Treatment. 2016; 57(59): 28715-28723.

33. Zhao R, Zhou Z, Zhao X and Jing G. Enhanced Cr(VI) removal from simulated electroplating rinse wastewater by amino-functionalized vermiculite-supported nanoscale zero-valent iron*.* Chemosphere. 2019; 218: 458-467.

34. Wang C, Wu Y, Qu T, Liu S, Pi Y, and Shen J. Enhanced Cr(VI) removal in the synergy between the hydroxyl-functionalized ball-milled ZVI/Fe3O4 composite and Na2EDTA complexation*.* Chem Eng J. 2019; 359: 874-881.

35. Huang X, Wang Y, Li X, Guan D, Li Y, Zheng X, et al. Autocatalytic Decomplexation of Cu(II)–EDTA and Simultaneous Removal of Aqueous Cu(II) by UV/Chlorine*.* Environ Sci Technol. 2019; 53(4): 2036-2044.

36. Hernandez-Martinez G R, Ortiz-Alvarez D, Perez-Roa M, Urbina-Suarez N A and Thalasso F. Multiparameter analysis of activated sludge inhibition by nickel, cadmium, and cobalt*.* J Hazard Mater. 2018; 351: 63-70.

37. Kapoor V, Elk M, Li X, Impellitteri C A and Santo Domingo J W. Effects of Cr(III) and Cr(VI) on nitrification inhibition as determined by SOUR, function-specific gene expression and 16S rRNA sequence analysis of wastewater nitrifying enrichments*.* Chemosphere. 2016; 147: 361-367.

38. Mishra A and Malik A. Simultaneous bioaccumulation of multiple metals from electroplating effluent using Aspergillus lentulus*.* Water Res. 2012; 46(16): 4991-4998.
